# Screening of Material Defects using Universal Machine‐Learning Interatomic Potentials

**DOI:** 10.1002/smll.202503956

**Published:** 2025-08-03

**Authors:** Ethan Berger, Mohammad Bagheri, Hannu‐Pekka Komsa

**Affiliations:** ^1^ Microelectronics Research Unit, Faculty of Information Technology and Electrical Engineering University of Oulu P.O. Box 4500 Oulu FIN‐90014 Finland; ^2^ Department of Physics Chalmers University of Technology Gothenburg SE‐41296 Sweden; ^3^ Nanoscience Center Department of Physics University of Jyväskylä Jyväskylä Finland

**Keywords:** 2D materials, benchmark, defects, machine‐learning interatomic potential, vacancies

## Abstract

Finding new materials with previously unknown atomic structure or materials with optimal set of properties for a specific application greatly benefits from computational modeling. Recently, such screening has been dramatically accelerated by the invent of universal machine‐learning interatomic potentials that offer first principles accuracy at orders of magnitude lower computational cost. Their application to the screening of defects with desired properties or to finding new stable compounds with high density of defects, however, has not been explored. Here, it is shown that the universal machine‐learning interatomic potentials have reached sufficient accuracy to enable large‐scale screening of defective materials. Vacancy calculations are carried out for 86,259 materials in the Materials Project database and the formation energies analyzed in terms of oxidation numbers. The application of these models is further demonstrated for finding new materials at or below the convex hull of known materials and for simulated etching of low‐dimensional materials.

## Graphical abstract

The accuracy of universal machine‐learning interatomic potentials for defect screening are benchmarked and found to yield sufficiently reliably results for vacancies. High‐throughput vacancy formation energy calculations are carried out for 86,259 materials. Formation energies are found to be strongly correlated with oxidation numbers and can also be used to simulate etching of 2D materials in acidic solution.

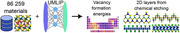

## 1 Introduction

Computer simulations of materials based on first principles methods, quantum chemistry, and density‐functional theory (DFT), are playing an increasingly important role in the discovery of new materials. On one hand, materials with optimal properties for specific applications can be found by carrying out explicit high‐throughput calculations or employing databases of previously calculated data.^[^ ^1^, ^2^ ^]^ These predictions can then be experimentally verified, as demonstrated in the case of magnetic materials,^[^ ^3^ ^]^ high‐entropy alloys,^[^ ^4^ ^]^ thermoelectrics,^[^ ^5^ ^]^ and dielectrics,^[^ ^6^ ^]^ to name a few. On the other hand, new stable materials, i.e., falling below the convex hull of all previously known stable compounds, can be discovered by atom substitution,^[^ ^7^ ^]^ crystal structure prediction,^[^ ^8^ ^]^ simulated etching,^[^ ^9^ ^]^ or random structure search.^[^ ^10^, ^11^ ^]^

In recent years, the progress in materials research has been massively accelerated by the development of machine‐learning (ML) methods and in the context of atomic simulations machine‐learning interatomic potentials (MLIPs), which can yield results nearly matching the accuracy of first‐principles calculations at a fraction of the computational cost.^[^ ^12^, ^13^ ^]^ Going further, to eliminate the burden of training application‐specific MLIPs, universal MLIPs (UMLIP) have been developed with the intention to work for any system with any elements. These are trained on the massive datasets collected from Materials Project (MP),^[^ ^14^ ^]^ Alexandria,^[^ ^15^ ^]^ or Open catalyst project.^[^ ^16^ ^]^ Over the last three years only, many competing UMLIPs have been reported, such as M3GNet,^[^ ^17^ ^]^ CHGNet,^[^ ^18^ ^]^ ALIGNN,^[^ ^19^ ^]^ MACE,^[^ ^20^, ^21^ ^]^ GNoME,^[^ ^10^ ^]^ MatterSim,^[^ ^22^ ^]^ EquiformerV2,^[^ ^23^ ^]^ ORB,^[^ ^24^ ^]^ and SevenNet.^[^ ^25^ ^]^ The leading universal potentials are showing few tens of meV accuracy on formation energies of stable compounds,^[^ ^26^ ^]^ which is comparable to the energy differences observed near the convex hull and thus the modern UMLIPs are highly promising for accelerated screening of new stable materials.

It is then somewhat surprising that the applicability of UMLIPs for defect screening has not been properly benchmarked to date. This may be due to the fact that there does not exist a similar large dataset of defect calculations, although several smaller databases targeting a set of materials or a set of defects have been collected.^[^ ^9^, ^27^, ^28^, ^29^, ^30^, ^31^, ^32^, ^33^ ^]^ The training sets for UMLIPs do not explicitly contain defective materials, although motifs resembling their atomic structures might still be present in the training set. Nevertheless, this is expected to lead to lower accuracy for defective systems. Fortunately, screening defects is also more forgiving as they often exhibit a large range of values up to 10 eV. For finding the defects with lowest formation energy, determining whether they are likely to be present in high concentration under given conditions (given a choice of chemical potentials), or if the formation energies are negative which entails material decomposition, accuracy of hundreds of meVs can be sufficient.^[^ ^34^, ^35^, ^36^ ^]^

In this paper, we start by benchmarking UMLIPs for defect calculations by collating results from several existing targeted defect databases. As the UMLIPs are found to exhibit accuracy sufficient for defect screening, we carry out further analysis. We first calculate vacancy formation energies for all materials in the Materials Project database and analyze the trends in terms of oxidation numbers. We then demonstrate how these calculations can be used to find new materials near the convex hull, either stable compounds or materials that are likely to host a large concentration of vacancies. Finally, we discover new 2D materials via simulated non‐equilibrium etching from stable parent phases.

## 2 Results

### 2.1 Benchmark of UMLIPs

In order to assess the accuracy of UMLIPs for the prediction of defect formation energy, we tested four different UMLIPs, namely MACE,^[^ ^21^ ^]^ CHGNet,^[^ ^18^ ^]^ M3GNet^[^ ^17^ ^]^ and ALIGNN.^[^ ^19^ ^]^ These four universal potentials have the advantage of being trained using a similar training set based on the Materials Project database.^[^ ^14^ ^]^ All four UMLIPs are tested and compared using three existing defect databases by Angsten et al.,^[^ ^27^ ^]^ Huang et al.,^[^ ^28^ ^]^ and Björk et al.^[^ ^9^ ^]^ These datasets were chosen because they cover a large variety of materials and because they have been calculated using the same PBE functional^[^ ^37^ ^]^ as used for training the above‐mentioned UMLIPs, and thus we can rule out any error contributions arising from the choice of functional. The dataset of Angsten et al. contains vacancies of face‐centered cubic (FCC) and hexagonal close‐packed (HCP) structures for a large part of the periodic table.^[^ ^27^ ^]^ The dataset of Huang et al. contains substitutional and vacancy defects in 2D materials, such as h‐BN, MoS_2_, GaSe, and BP.^[^ ^28^ ^]^ The dataset of Björk et al. contains vacancies for 77 layered transition metal dichalcogenide (TMDC), MAX, and YRuSi‐type phases.^[^ ^9^ ^]^ Note that all three datasets considered here do not include charged defects, and therefore all benchmarked defects are neutral. Given that UMLIPs are also trained only on neutral bulk systems, we believe that the predicted defect formation energies would also predominantly correspond to the neutral charge state.

While the contents of each dataset differ on the structural information given (relaxed, unrelaxed, or no structure) and how the energies are reported (total energy, formation energy and the choice of chemical potentials), our UMLIP calculations are carried out in a way that is consistent with each dataset. Further details concerning how each dataset was processed are given in the Supporting Information.

**Figure 1:**
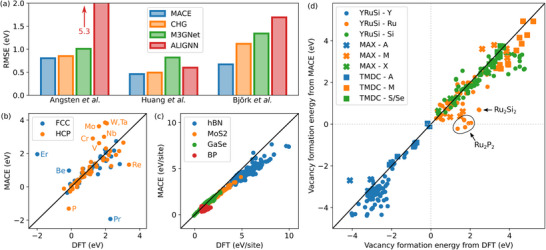
a) Root‐mean‐squared error of the UMLIPs tested on data sets of Angsten et al., Huang et al., and Björk et al. (Refs. [27, 28], and [9], respectively). b–d) Comparison of the defect formation energy from MACE and DFT for b) Angsten et al., c) Huang et al., and d) Björk et al. datasets.

Benchmarking results are presented in **Figure** **1**. In particular, Figure 1a shows the root mean square error (RMSE) over all three dataset for all four UMLIPs. MACE performs best on all datasets, with RMSE values of 0.80, 0.46, and 0.67 eV for datasets from Angsten et al., Huang et al., and Björk et al., respectively. Previous benchmarking of UMLIPs for other material properties have also found MACE to extrapolate well to previously unencountered systems.^[^ ^20^, ^38^, ^39^ ^]^ CHGNet and M3GNet are also found to reasonably predict defect formation energies, although they show larger errors for the layered materials included in the dataset of Björk et al. On the contrary, ALIGNN seems to perform poorly, which could be explained by the large errors in the chemical potentials, see Figure S13 (Supporting Information).

More detailed comparison of the MACE and DFT results are given in the correlation plots in Figure 1b–d. Similar plots for the other UMLIPs can be found in the Supporting Information (see Figures S1, S2, and S3, Supporting Information). While the agreement for the dataset of Angsten et al. (Figure 1b) is very good for most elements, a few data points are poorly predicted by MACE. For example, HCP phosphorus and FCC erbium and praseodymium show large errors, which are also found when using other UMLIPs (see Figure S1, Supporting Information). Additionally, transition metals such as molybdenum, tungsten and tantalum also show relatively large errors in the HCP structure, which is not their optimal structure and likely explains the poor performance with MACE. For the 2D materials, Figure 1c, MACE accurately predicts the defect formation energy at low energies. Low formation energy means that the local structural environment is chemically stable and consequently it is likely that similar local environments were already present in the training set for UMLIPs. While the agreement somewhat worsens at high formation energy, the dependence is still linear, which is important in material screening. Largely similar behavior can be observed in Figure 1d for the layered bulk phases included in the database of Björk et al. In this previous work, the goal was to identify which atoms have a negative vacancy formation energy in acidic solutions and could therefore be etched. In Figure 1d, etched atoms are shown in blue and all have Δ*F*_*v*_ < 0 according to DFT calculations. It is clear that MACE correctly makes the separation between etched and non‐etched atoms, with only a few structures containing ruthenium being wrong. Note that other UMLIPs struggle with the same materials, suggesting that the problem arises from the insufficient training set for these compounds and not from the ML model.

We also carried out benchmarking for datasets including other types of defects, such as substitutions and interstitial atoms, reported in Refs. [40] and [30] (see Figures S4 and S5, Supporting Information). While the agreement between UMLIPs and DFT calculations remain reasonable for substitutions, it gets clearly worse for interstitial defects. For this kind of defect, DFT formation energy can go up to 20 eV and such highly unstable environments are unlikely to be present in the MP database, resulting in inaccurate predictions from UMLIPs. A summary of the RMSE of the four UMLIPs over all datasets is given in the Supporting Information (see Table S1, Supporting Information).

Overall, we find that the UMLIPs, and MACE in particular, are successful in predicting vacancy formation energies for a diverse set of materials. In particular, MACE is found to give the most accurate predictions, with RMSE between 0.4 and 0.8 eV for the three studied datasets. These values are comparable to the accuracy of specialized ML models designed to predict defect formation energies in a limited set of defects/materials: 0.5–1 eV for oxide defects,^[^ ^32^ ^]^ 0.4 eV for neutral oxygen vacancies,^[^ ^41^ ^]^ 0.45–0.7 eV for vacancies in oxide perovskites,^[^ ^42^ ^]^ 0.67 eV for TMDCs,^[^ ^43^ ^]^ 1 eV for a larger set of defects and materials,^[^ ^44^ ^]^ 0.27–0.44 eV for vacancies in oxides.^[^ ^31^ ^]^

### 2.2 High‐Throughput Vacancy Calculations

**Figure 2:**
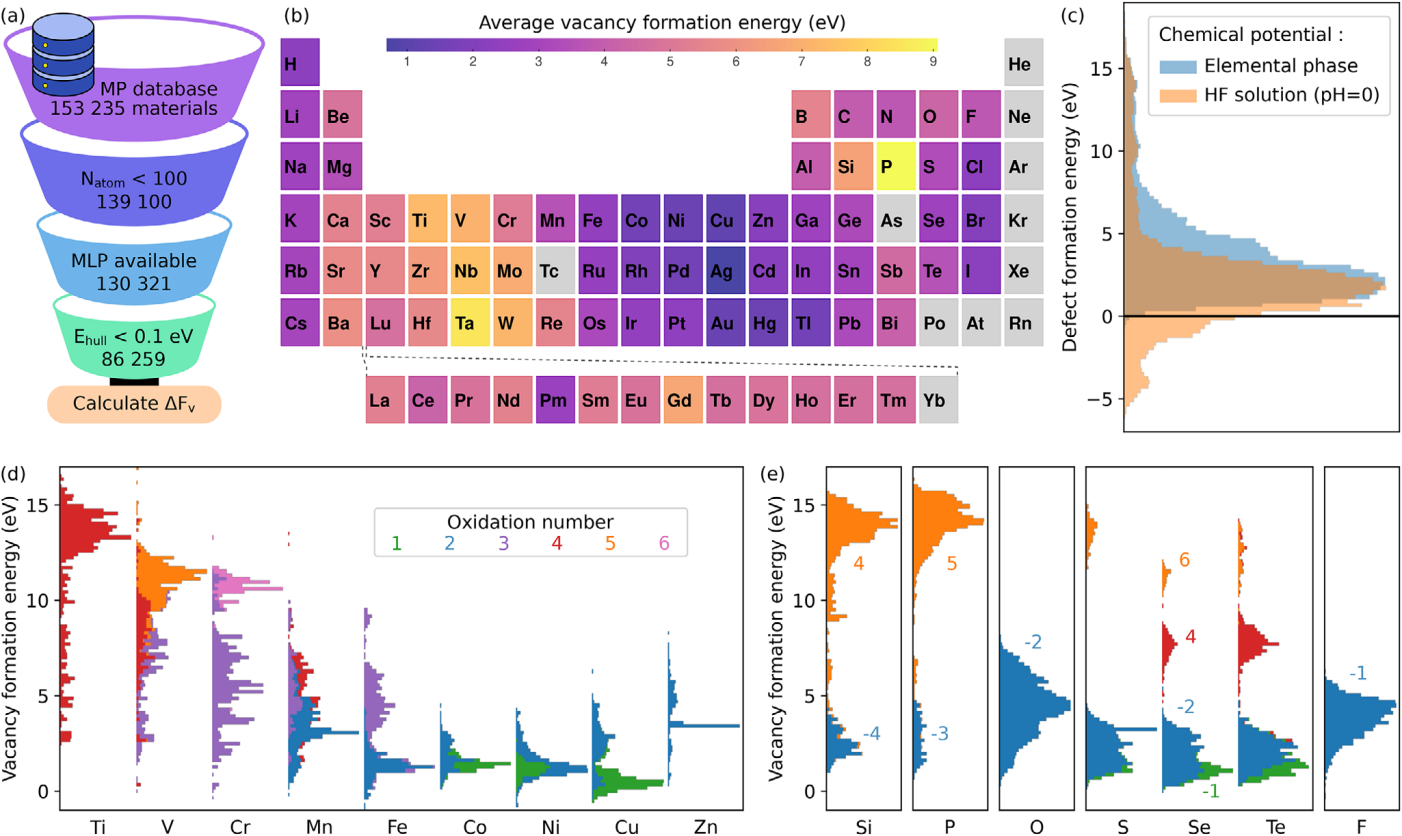
a) Materials project (MP) database screening procedure. The number of structures in each step is indicated. b) Average vacancy formation energy for every element in the periodic table. c) Histograms from all vacancy formation energy calculations with two different choices of chemical potentials. d,e) Stacked histograms of the vacancy formation energies for the dominant oxidation states of a few selected elements. In panels (b), (d), and (e), the vacancy formation energies are obtained using elemental chemical potentials.

With MACE found to provide reliable vacancy formation energies, we proceeded to calculate vacancy formation for most materials in MP database subject to selected screening conditions. As illustrated in **Figure** **2**a, we limit our study to materials with less than 100 atoms in the unit cell, (meta)stable materials corresponding to energy above convex hull less than 0.1 eV/atom, and excluding noble gases, arsenic, technetium, promethium, ytterbium, and elements heavier than Bi (not included in MACE). This screening still resulted in a total of 86,259 materials. At this stage, we only calculated the unrelaxed vacancy formation energy due to i) computational cost, ii) the ease of analysis, since in some cases relaxation might dramatically alter the structure, and iii) it provides an upper bound to the formation energy as relaxation can only lower the formation energy.

Figure 2b shows the average vacancy formation energy over the periodic table. The lowest formation energies are found for alkali metals, noble metals, and heavier halogens. One generally expects high formation energies with high oxidation state and strong chemical bonds. Here, we carry out analysis based mainly on the oxidation state, which is fairly straightforward to extract and a convenient atom‐specific scalar quantity. Noble metals, by definition, do not like to form compounds, and thus low vacancy formation energies are expected. Alkali metals and halogens have oxidation state ±1, often present as singly charged ions that are relatively easy to extract from the host lattice. Group‐11 metals (Cu, Ag, and Au) have a full *d*‐shell and a single *s*‐electron, and thus they behave largely similarly to alkali metals and with similar average formation energies. On the contrary, high formation energies are found for refractory metals, maximum found for Ta, and also *p*‐block elements, with Si and P particularly standing out.

In some cases, the formation energy distribution is multimodal, and thus average formation energy is not sufficiently descriptive. Distributions for few selected elements, and further grouped by their oxidation states, are shown in Figure 2d,e. In Table S2 (Supporting Information), we list the proportion of oxidation states in the database and in Table S3 (Supporting Information) the average formation energies for all elements at all oxidation states. Oxygen and fluorine both show a very wide distribution, but only existing in the −2 and −1 oxidation state, respectively. Si, on the other hand, shows a clear bimodal distribution, dominated by +4 and −4 oxidation states. The average formation energy for the +4 oxidation state is very high at 12.6 eV. A large proportion of these arise from silicates with SiO_4_ units, in which removing a silicon atom leaves four undercoordinated O atoms and resulting in high formation energy. Such high formation energies are not seen in the O distribution, since, while the Si‐O bond strength is the same, removing O atom results in only one or two undercoordinated Si atoms and, consequently, the formation energy is also lower by a factor of 2–4. The low formation energy peak arises from −4 oxidation state, often associated with Si atom surrounded by metals with high coordination numbers (CN). In Figure S6 (Supporting Information), we show the distributions grouped by CN, which verifies that the high‐energy peak is dominated by four‐coordinated Si atoms, while the low‐energy peak consists of high‐CN Si (largest fraction for CN of 9).

Similar findings apply to P, where bimodal distribution with peaks arising from oxidation states +5 and ‐3 are observed. The high formation energy peak at 13.7 eV with +5 oxidation state arises mainly from ${\mathrm{PO}}_{4}^{3-}$ units, quantitatively similar to the case of Si, whereas an example of −3 oxidation state is phosphine PH_3_. Figure S6 (Supporting Information) shows that the high‐energy peak corresponds to 4‐coordinated P and 3‐coordinated P have very low formation energies. Chalcogen elements S, Se, and Te show particularly clear grouping with the oxidation state. These allow us to extract reasonably representative oxidation‐state dependent average formation energies. For example, selenium has an average vacancy formation energy of 2.2, 7.5, and 11.1 eV in oxidation states ‐2, +4 and +6, respectively. Detailed results for all elements and all oxidation states can be found in Table S3 (Supporting Information).

In Figure 2d, we show the distributions from the 3*d* transition metal series. As mentioned, Cu is found primarily at +1 oxidation state, with a narrow distribution (average 0.6 eV). While heavy atoms between Co and Zn only show oxidation number of +1 and +2, lighter elements can adopt higher oxidation states. In few cases, a clear grouping with the oxidation state can be observed, such as Cr^+3^/Cr^+6^, Fe^+2^/Fe^+3^ or Cu^+1^/Cu^+2^. Overall, higher oxidation states are found to yield higher formation energies. Transition metals from the 4d and 5d series are shown in Figure S7 (Supporting Information), with largely similar trends. In addition to the oxidation number, vacancy formation energies are also found to depend on the neighboring elements (see Figure S8, Supporting Information). Additionally, distributions with CN are given in Figure S9 (Supporting Information), but show no obvious grouping.

Overall our results indicate a fairly strong correlation between the oxidation state of the element and its vacancy formation energy. This hints at the possibility of using the oxidation state as a descriptor in carrying out defect screening. Moreover, one can use the data in Table S3 (Supporting Information) as a first approximation for an unknown vacancy formation energy.

### 2.3 Ordered Vacancy Compounds below Convex Hull

In the total formation energy distribution, Figure 2c, one can distinguish a part of the distribution extending to negative energies for 332 materials. This would correspond to spontaneous defect formation (when kinetically allowed) until equilibrium is reached by e.g., adopting a stable non‐zero vacancy concentration or transforming to another phase. In other words, these cases hint for a possibility to find new materials that are below the convex hull defined by the materials in the MP database. However, for more quantitative assessment, we need to include three ingredients: exploration of different vacancy concentrations and their ordering (configurations), structural relaxations, and energy comparison to the convex hull.

Because of the errors from MACE, the stability of vacancies with formation energies close to zero cannot be predicted confidently. In our case, any structure with vacancy formation energy between −0.75 and 0.75 eV is not considered. The value 0.75 eV is chosen to match the average RMSE of MACE over the benchmarked datasets. Out of the 332 materials with negative formation energy, 34 were found to have vacancy formation energies below −0.75 eV.

**Figure 3:**
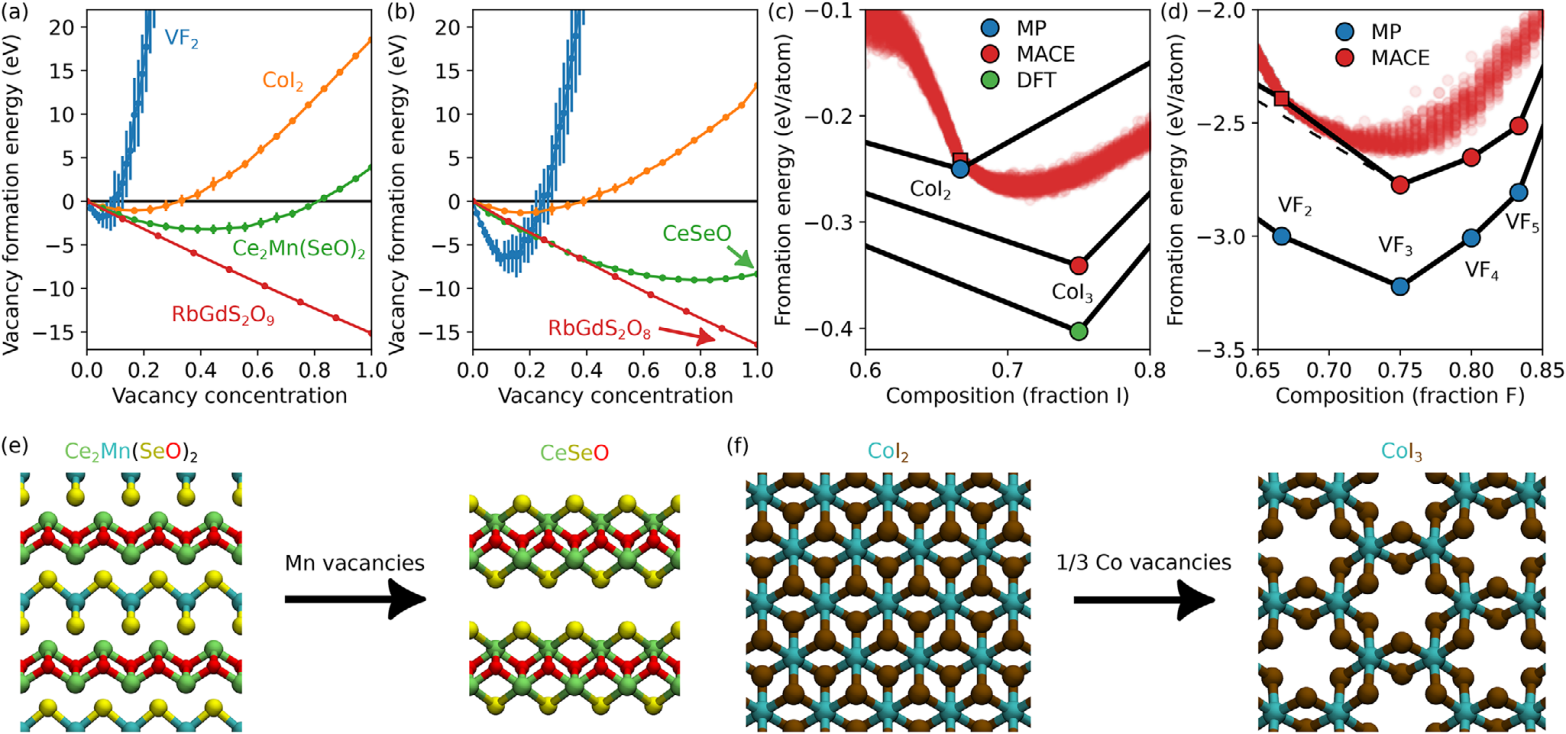
a) Unrelaxed defect formation energy with respect to the vacancy concentration for a few selected materials. b) The formation energies after accounting for the structural relaxations. c–d) Convex hull of Co‐I and V‐F systems, respectively. In panels (c) and (d), the red dots show the formation energies of structures containing defects. In panels (a–d), the vacancy formation energy is calculated using elemental chemical potentials. e–f) Balls‐and‐sticks representations of Ce_2_Mn(SeO)_2_ and CoI_2_, respectively. Initial structures from MP are compared with structures with minimal formation energy after removing atoms with negative vacancy formation energies.

In **Figure** **3**a, we show the unrelaxed formation energies as a function of defect concentration for four selected materials that were indicated to have negative formation energy and which show qualitatively different behavior. Except for VF_2_, the deviation of energies around the mean is still small, indicative of weak defect‐defect interactions. VF_2_, CoI_2_, and Ce_2_Mn(SeO_2_)_2_ show minimum formation energy at increasing concentration, with the vacancies created in the V, Co, and Mn sublattices, respectively. One of the inequivalent O atoms in RbGdS_2_O_9_ is very weakly bound and all are expected to be removed (100% vacancy concentration in Figure 3 referring only to the relevant sublattice), suggesting a possible problem in the experimentally determined structure.

In Figure 3b, the same results are shown after relaxation of all relevant systems (including the pristine host). One would expect formation energies to decrease and this is indeed found for all cases, although clearly more pronounced in the cases of VF_2_ and Ce_2_Mn(SeO_2_)_2_. Consequently, the vacancy concentration yielding the minimum formation energy increased, from 6 to 14% in the case of VF_2_ and from 44 to nearly 100% in the case of Ce_2_Mn(SeO_2_)_2_. The structure of Ce_2_Mn(SeO_2_)_2_ before and after etching all Mn atoms are illustrated in Figure 3e. Interestingly, the structure might be intuitively (or by robocrystallographer)^[^ ^45^ ^]^ thought to consist of Ce_2_O_2_ and MnSe_2_ layers and thus the possibility to etch Mn atoms would be easily missed.

Having found the lowest energy structures for the defective systems, we can next map their energy with respect to the convex hull. We emphasize that while the choice of elemental phase references used here is fairly stringent in the sense that it leads to high formation energies, negative defect formation energy does not necessarily imply that the defective phase would be below the convex hull. In fact, the defect formation energy can be directly compared to the slope of the convex hull, and the defective structure falls below the convex hull only if the defect formation energy is lower than the slope of the convex hull. Co–I and V–F systems both fall into this category. The relevant part of the phase diagram of Co–I is shown in Figure 3c. MP convex hull contains only CoI_2_ with the MACE closely reproducing its formation energy. The calculated systems with Co‐vacancies (I fraction above 2/3) fall under the convex hull for a wide range of vacancy concentrations. It turns out, however, that the underlying reason for this result is that MP database does not include the stable CoI_3_ phase (represented in Figure 3f), although it has been previously predicted computationally by substitution of atoms with chemically similar ones in known lattices.^[^ ^7^, ^46^ ^]^

In the case of V–F, Figure 3d, the convex hull in MP consists of VF_2_, VF_3_, VF_4_, VF_5_ phases. When recalculated with MACE, the VF_2_ phase ends up falling slightly above convex hull. As the VF_3_ phase is clearly below VF_2_, the defective system formation energies end up falling very close to the convex hull up to about VF_2.5_, and even slightly below convex hull for some configurations when compared to the VF_2_–VF_3_ line. Thus we think it should be possible to synthesize VF_2_ with a large range of vacancy concentrations. A more complete exploration of the possible ordered vacancy compounds would require systematic exploration of all possible supercells and vacancy configurations, and while UMLIPs can accelerate this process, it is beyond the scope of this paper.

For multielement compounds the convex hull is multidimensional and thus difficult to visualize. However, the formation energy can still be easily compared with the convex hull of competing phases. CeSeO is located on the CeSe_2_–CeO_2_ line and is found to be 0.14 eV/atom below the convex hull. Similarly, RbGdS_2_O_8_ has a formation energy 0.23 eV/atom below plane made of Rb_2_S_2_O_7_, Gd_2_O_3_, and SO_3_. A structure somewhat similar to CeSeO was found by chemical substitution,^[^ ^7^ ^]^ while we are not aware of previous reports for RbGdS_2_O_8_. Thus, these materials could be examples of stable materials below MP convex hull.

Overall, we have demonstrated how a defect screening can be used to identify known stable materials missing from the database (CoI_3_), possible new stable materials (CeSeO and RbGdS_2_O_8_), as well as systems that are likely to host ordered vacancy compounds (V–F). Complete results for all 34 materials with vacancy formation energies below −0.75 eV are presented in Figure S10 and Table S4 (Supporting Information). In total, 20 materials are found to have negative formation energy at 100% defect concentration. Interestingly, some materials were also found to show two distinct formation energy minima at different defect concentrations of 25% and 100%, corresponding to BrO_4_ units replaced by BrO_3_ or Br, respectively. These are discussed in more details in the Supporting Information.

### 2.4 2D Materials from Simulated Etching

Another application of vacancy formation energy calculations is the study of chemical etching of bulk phases into low dimensional materials, as previously shown by studying etching of MXenes layers from MAX phases^[^ ^36^ ^]^ and later extended to other layered phases.^[^ ^9^ ^]^ Here, we look for new 2D layers by screening the whole database of vacancy formation energies in a way similar to Björk et al.^[^ ^9^ ^]^

**Figure 4:**
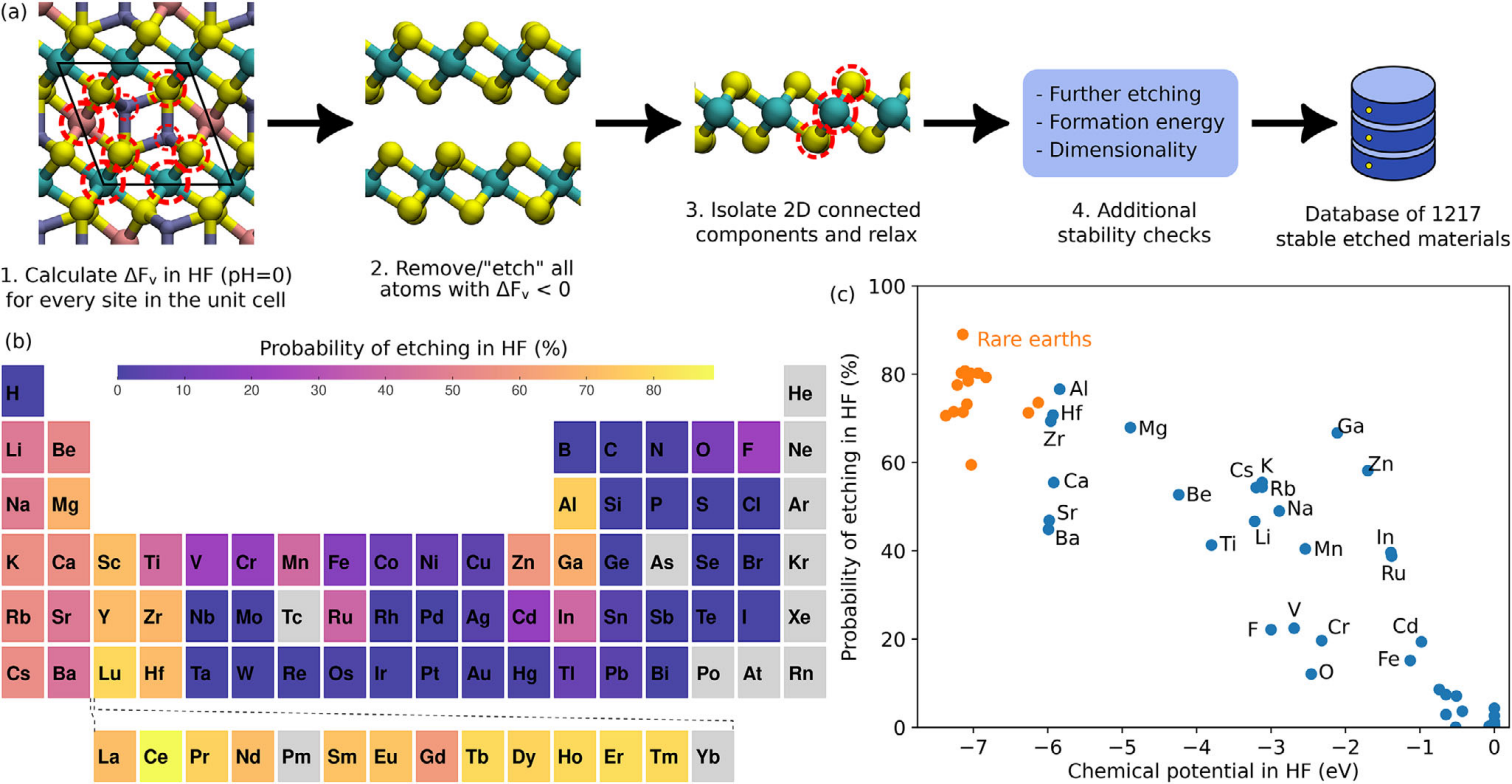
a) Workflow for the identification of potential 2D layer materials from chemical etching. Dashed red circles indicate the sites in the unit cell for which defect formation energy is calculated. b) Calculated probability of chemical etching in HF for each elements of the periodic table. Excluded elements are shown in gray. c) Probability of etching with respect to the chemical potential in HF. In panels (b) and (c), the probability of etching is obtained from the vacancy formation energies using chemical potentials in HF.

A schematic of the workflow is represented in **Figure** **4**a. Starting from the vacancy formation energy database, atoms with negative vacancy formation energy are removed from the atomic structures, with the chemical potentials corresponding to conditions in aqueous HF solution at pH = 0. As can be seen from the histogram in Figure 2c, a large portion of materials will have one or more elements etched. By considering the whole database, one can obtain the etching probability for each element, which is presented in Figure 4b. Few groups of high etching probability stand out, mainly consisting of typical cation elements. For all these elements, the high etching probability can be explained by a low chemical potential in HF solution, as illustrated in Figure 4c. On the contrary, elements with a high chemical potential are rarely etched. Examples would be some transition metals (such as niobium, molybdenum, tantalum, and tungsten) and chalcogens other than oxygen. As a result, many of the etched layers presented later contain these elements.

To find chemically etched 2D materials, layers are first isolated and their atomic structure is relaxed. This procedure resulted in a total of 4370 candidate layers, which are further screened to identify the best candidates. Next, the vacancy formation energies of the layers (denoted $\Delta F_{v}^{\left(2\right)}$, (2) referring to “second round” of etching) are computed. The condition $\Delta F_{v}^{\left(2\right)}>0$ ensures that none of the atoms within the layer is etched. In some cases, relaxing a layer after etching lead to the formation of 1D or 0D clusters. To avoid such cases, the dimensionality of the relaxed layers is calculated again and we only consider those with a 2D score higher than 0.9. Additionally, layers with positive formation energy are also not considered. After applying these three conditions, we find 1217 candidate layers. In order to identify the best candidates, we define the highest etched element formation energy (HEE) as well as the lowest non‐etched element formation energy (LNE) for each layer. Best candidate layers have a low HEE and a high LNE, as well as a high defect formation energy $\Delta F_{v}^{\left(2\right)}$. The database is therefore further screened by applying HEE < −0.75 eV, LNE > 0.75 eV and $\Delta F_{v}^{\left(2\right)}>0.75$ eV, resulting in a total of 259 layers labeled as “best candidates”.

**Figure 5:**
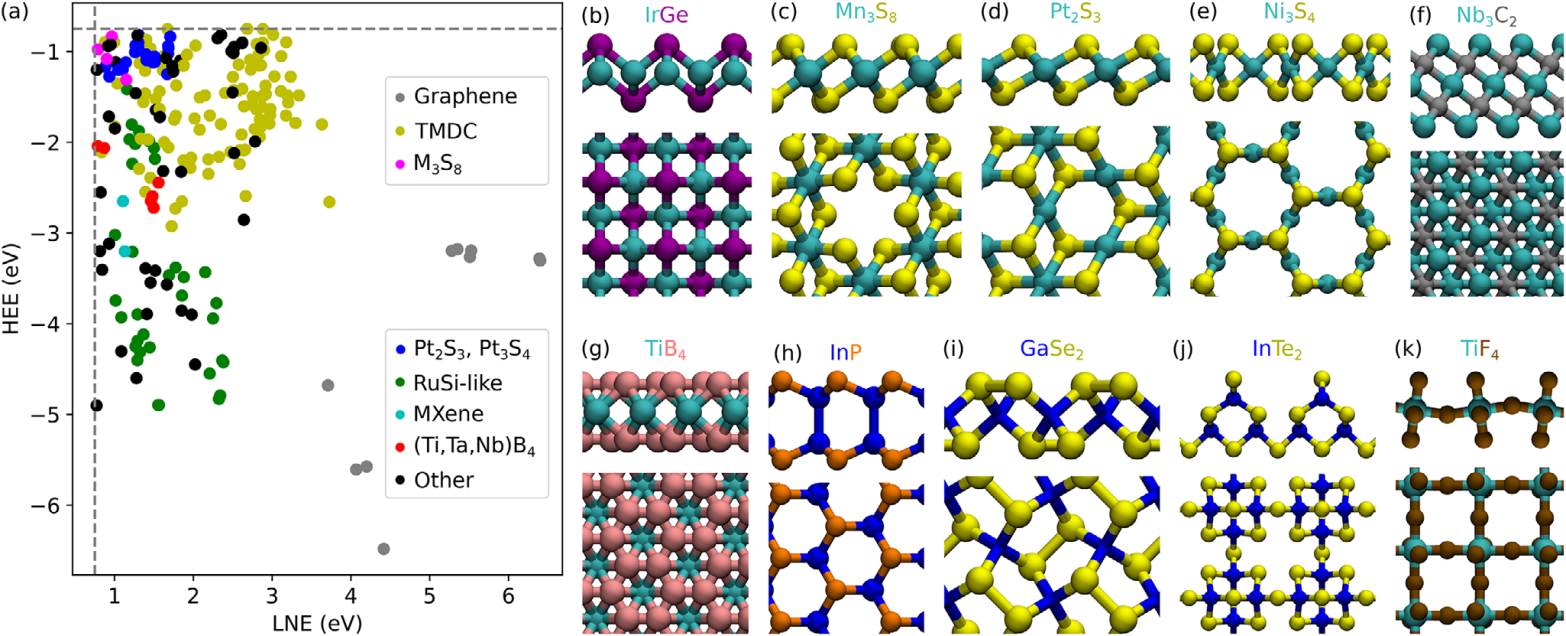
a) Comparison between the highest etched element (HEE) and lowest non‐etched element formation energies (LNE) for binary layers. Vacancy formation energies are calculated using chemical potentials in HF. Note that the plot only shows the region (HEE < −0.75, LNE > 0.75). Groups of layers with similar atomic structure are indicated by coloring. b–k) Representation of a few of the best candidate binary layers. Top panels show side view of the layer, while bottom panels show the top view. The color coded formula is given for each structure.

**Figure** **5** presents the best candidate unaries and binaries (see Table S5, Supporting Information for a complete list of all layers). In particular, Figure 5a shows the HEE and LNE of these layers. Many known materials are recovered. For example, graphene is found to have the highest LNE, as well as the lowest HEE values depending on the initial structure, such as LiC_6_ and EuC_6_, where lithium and europium are respectively etched. Note that graphene is also the material with the highest value for $\Delta F_{v}^{\left(2\right)}$. Among layers with low HEE, there are many with an atomic structure similar to RuSi recently found by Björk et al.^[^ ^9^ ^]^ Figure 5a also shows two MXene structures, namely Nb_3_C_2_ and Ta_3_C_2_ (see Figure 5f). Although these two MXenes have not been synthesized, there has been report of successful synthesis of other similar layers based on Nb and Ta.^[^ ^47^ ^]^

A large fraction of the best candidates are TMDCs, ranging from well known MoS_2_, NbS_2_, or TaSe_2_^[^ ^48^ ^]^ to more exotic RhSe_2_ or RhTe_2_. Interestingly, TMDCs can be obtained by etching a large variety of elements. For example, NbS_2_ can be found by etching alkali metals such as Li and Cs, early transition metals such as Ti and V or post‐transition metals such as In. In addition to simple TMDCs, we also find TMDC layers including defects, either metal or chalcogen vacancies. An example of metal vacancy would be Mn_3_S_8_ layers, which is represented in Figure 5c. Note that these layers are found by etching two different elements. For chalcogen vacancies, we find two different cases, namely Pt_2_S_3_ and Pt_2_Se_3_. An example of the atomic structure can be seen in Figure 5d. Similar ordered vacancies have already been experimentally realized in PtTe_2_.^[^ ^49^ ^]^ The final example of binary materials containing transition metals and chalcogens is Ni_3_S_4_, Pd_3_Se_4_, or Pt_3_S_4_. All these layers have a Kagome structure similar to the one shown in Figure 5e, and are obtained by etching alkali metals from a parent phase resembling Cs_2_Ni_3_S_4_. Previous experimental study have already highlighted the possibility to etch Cs atoms from Cs_2_Ni_3_S_4_, although they could not reach Ni_3_S_4_ monolayers.^[^ ^50^ ^]^

The screening also revealed semiconducting layers, such as InP (Figure 5h), which has already been experimentally synthesized using chemical etching^[^ ^51^ ^]^ and has the same atomic structure as the well known GaSe.^[^ ^52^, ^53^ ^]^ Although GaSe is not included in our results, a different form of gallium selenide is, namely GaSe_2_. This layer takes a pentagonal atomic structure similar to PdSe_2_^[^ ^54^, ^55^, ^56^ ^]^ and is represented in Figure 5i. Another new semiconducting layer is InTe_2_ (see Figure 5j), etched from CsInTe_2_. Although such parent phases are known to be present in alkali‐metal treated CIGS solar cells, there are no reports on isolating them as monolayers. Note that we also find other compositions with the same structure, such as InSe_2_ or GaTe_2_, as well as InTe_2_ in the pentagonal form (similar to GaSe_2_ in Figure 5i), although these examples do not meet the criteria to be among the “best candidates”.

Among the other lesser known materials, we first highlight TiB_4_, NbB_4_ and TaB_4_. These layers are composed of two hexagonal boron layers, with metal atoms located in‐between at the center of the hexagons (see Figure 5g). The parent phases are similar to bulk TiB_2_,^[^ ^57^ ^]^ but with alternating layers of Ti and either Zr or Hf, and the latter are etched to lead to TiB_4_. Thicker layers, such as Nb_2_B_6_ or Ta_3_B_8_, are also predicted. Note that these layers resemble MBenes, which are absent from our results due to terminations not being included. Finally, bulk materials such as CsTiF_4_ are found to be etchable to form TiF_4_. The atomic structure of this layer consist of corner sharing TiF_6_ octahedra (Figure 5k). Although very similar to other titanium fluoride structures,^[^ ^58^ ^]^ the stability of TiF_4_ in 2D form remains unknown.

**Figure 6:**
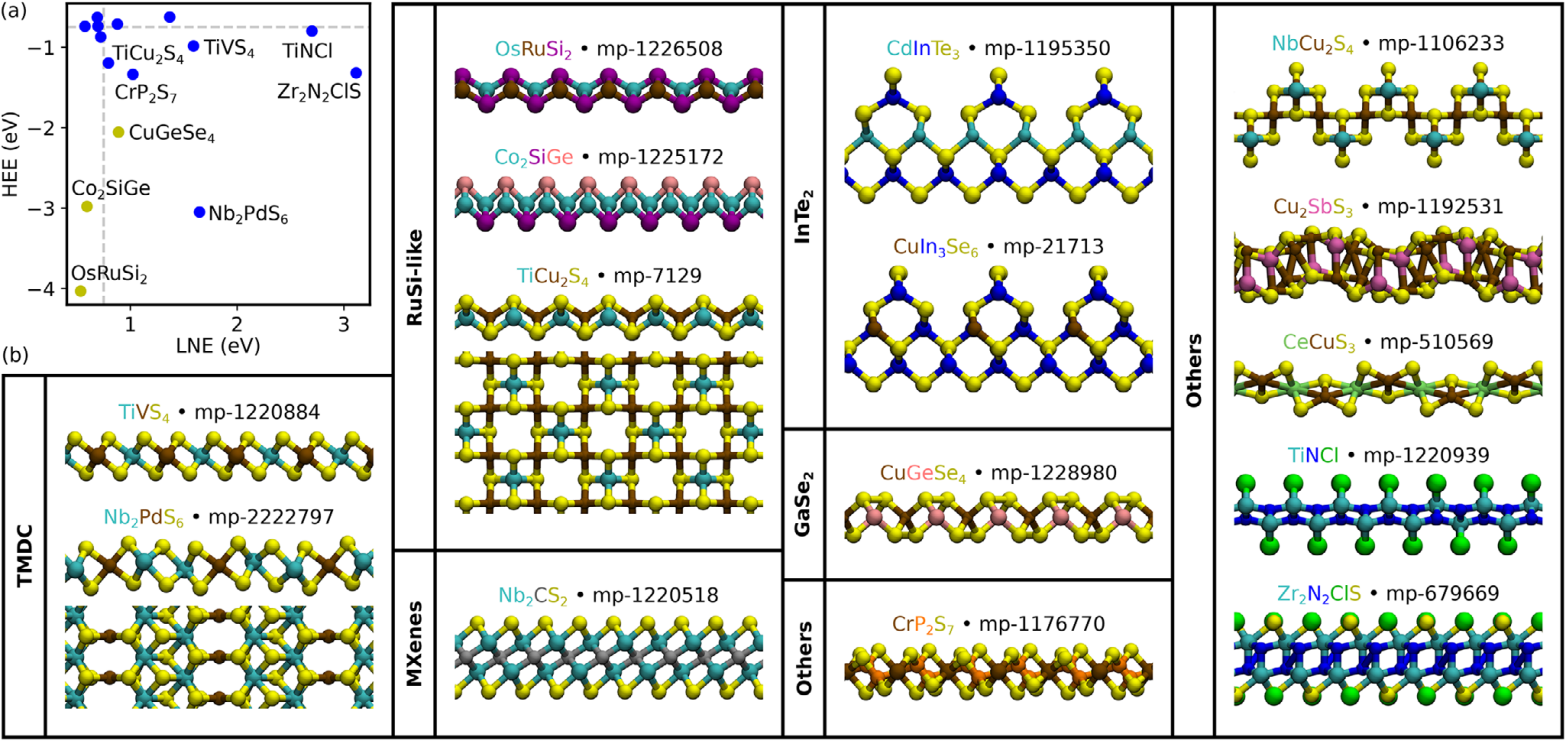
a) Comparison between LNE and HEE for ternary materials. Grey dashed lines show the limits for LNE = 0.75 and HEE = −0.75. Blue dots represent layers with $\Delta F_{v}^{\left(2\right)}>0.75$, while yellow ones have $0.5<\Delta F_{v}^{\left(2\right)}<0.75$. Vacancy formation energies are calculated using chemical potentials in HF. b) Representation of a few of the best candidate ternary layers. The structures are grouped depending on their resemblance with binary layers (see Figure 5 for the structure of binary layers). The color‐coded formula and the Materials Project ID (MPID) is given for each structure.

While previous works by Björk et al. only focused on binary layers etched from ternary parent phases, there was no need to limit the number of elements in our study due to the high performance of UMLIPs. Hence our calculations also predicted ternary (and quaternary) layers after etching, which are presented in **Figure** **6**. A complete list of ternary layers is available in Table S6 (Supporting Information). In particular, Figure 6a compares the LNE and HEE of the best candidates. Note that here, the threshold previously set at 0.75 is lowered to 0.5, resulting in a total of 77 layers (see Table S6, Supporting Information). The atomic structures of a few of the best candidate ternary layers are presented in Figure 6b. The dynamic stability of all ternary candidates is also tested by computing their phonon dispersions using MACE (see Figures S11 and S12, Supporting Information). Some of these structures are alloyed variants of the known binary structures, such as TMDC TiVS_4_. In Nb_2_PdS_6_, Nb has seven neighboring S atoms while Pd only has four, which leads to an atomic structure different from the usual TMDCs. We also find alloys of RuSi‐like layers, such as OsRuSi_2_ which has alternating elements on the Ru layer, or Co_2_SiGe which has a Janus structure. In addition to alloying, some showed ordered vacancies, such as TiCu_2_S_4_, with alternating Ti and Cu atoms and Ti vacancies on half of the sites. Two other similar structures were found, namely TiCu_2_Se_4_ and TiAg_2_S_4_.

Regarding MXenes, ternary layers Nb_2_CS_2_ and Ta_2_CS_2_ are included among the best candidates. These layers have the structure of MXenes with sulfur atoms as terminations and have already been synthesized, showing promising applications as superconductors.^[^ ^59^, ^60^ ^]^ Note that contrary to typical MXenes where aluminum atoms are etched, our calculation suggest that Nb_2_CS_2_ and Ta_2_CS_2_ can be obtained by etching 3d transition‐metals from V to Cu.

Ternaries similar to InTe_2_ and GaSe_2_ are also found. For the former, it has the same atomic structure as represented in Figure 5j but with an additional layer of InTe and alloying on the middle In sublattice. This leads to two different structures with different concentration of alloys, namely CdInTe_3_ and CuIn_3_Se_6_, which are both shown in Figure 6b. Regarding GaSe_2_, two similar pentagonal structures with alternating elements on the gallium site were found, namely CuGeSe_4_ and AgSnSe_4_.

In addition to ternaries resembling binary layers, our results also highlight ternary layers with completely new atomic structures, including CrP_2_S_7_, Cu_2_SbS_3_, or NbCu_2_S_4_. Note that for the latter, the same atomic structure is found for a wide variety of compositions, such as TaAg_2_Se_4_ or VCu_2_S_4_ (see Table S6, Supporting Information for a complete list). While rare earth metals are found to have a very high probability of being etch in HF, CeCuS_3_ is found to be among the candidates for chemical exfoliation. Other layers sharing the same atomic structure are also among candidates, although they have lower LNE and $\Delta F_{v}^{\left(2\right)}$ values, for example CoTmS_3_. In addition to rare earth metals, the same structure is also found in layers containing two transition metals, such as CuTiS_3_ and CuZrS_3_.

A majority of materials presented so far include chalcogens at the surface. This can be explained by their low probability of etching (see Figure 4b), which would tend to stabilize layers. Among ternary layers, we find two candidates with chlorine terminated surfaces, namely TiNCl and ZrNCl. These metal nitride halides are known to take two possible atomic structures, one cubic and one hexagonal,^[^ ^61^ ^]^ both represented in Figure 6b. Although nanosheets of ZrNCl have been synthesized by mechanical exfoliation,^[^ ^62^ ^]^ there is no report of chemical etching for this material. Note that for ZrNCl, our results also contain layers where half of the chlorine atoms are replaced by sulfur, leading to Zr_2_N_2_ClS. In particular, we find two structures with this formula, one with alternating Cl‐S atoms and one with a Janus type.

## 3 Discussion

In conclusion, calculations of vacancy formation energies contain useful information about materials properties, yet they are computationally demanding and usually restricted to a small amount of structures. We showed how UMLIPs can be used to drastically reduce the computational cost while maintaining a good accuracy. After carefully benchmarking four different UMLIPs, MACE was found to yield the most accurate predictions. It was then used to compute vacancy formation energies of 86,259 materials, giving access to large statistics for most elements in the periodic table. As an application, vacancy formation energies were used to explore new ordered vacancy compounds near the convex hull and the synthesis of new 2D layers using chemical etching. Most the ordered vacancy compounds below or near convex hull identified in this work were indicative of known compounds missing from the databases. Nevertheless, our work brings forth the importance of defects in the exploration of new materials, a dimension that has been previously largely ignored. That said, given the small energy differences near the convex hull, a more quantitative identification would benefit from more accurate UMLIPs, especially when defects other than vacancies are considered. We hope that this is achievable in the future, given the fast progress in the development of UMLIPs. At the same time, we recommend including defective materials in the training process to further accelerate their use in defect screening. At a small scale, for specific defects in a small set of materials, fine‐tuning of the foundation models can be a promising approach, but the effective fine‐tuning strategies for defective systems still need to be explored further.

Making use of the high efficiency of UMLIPs, this work predicted 1217 2D layers obtainable through chemical etching. These candidate layers include well known materials, as well as new exciting ones. Importantly, our approach could be readily applied to materials with any number of elements, any number of atoms in the unit cell, any crystal symmetry, and to cases where several elements may be etched. It is important to note that the current work did not investigate the stability of these layers, nor tested their experimental synthesis. Additional work is therefore required in order to validate some of the current predictions, as well as identify the properties of these new layers and their potential future applications. While surface terminations play an important role in the stability of some 2D layers, they have not been considered in this work. Including terminations would lower the formation energy and the vacancy formation energy of 2D layers. As a result, more layers would pass the screening depicted in Figure 4a. For example, Ti_3_C_2_ is a well known experimentally synthesized MXene layer, but not considered in this work because of the negative vacancy formation energy of titanium atoms. Testing various elements at different surface sites for a large number of structures is not straightforward and would prove challenging, hence the decision not to include terminations in the current work. The database containing etched layers could however be a good starting point for future studies on surface terminations of 2D layers. Additionally, general trends can also be observed using the large statistics available from MP materials. For example, Figure 4b shows which elements are most likely to be etched, and which ones are more stable in HF. These results can be further used for the design of new low‐dimensional materials and their parent bulk phase outside of the MP database.

## 4 Experimental Section

### Universal Machine‐Learning Interatomic Potentials

Four different UMLIPs were used, namely MACE^[^ ^21^ ^]^ (MACE‐MP‐0, small model size, without dispersion), CHGNet^[^ ^18^ ^]^ (default model from the chgnet python package), M3GNet^[^ ^17^ ^]^ (M3GNet‐MP‐2021.2.8‐PES model), and ALIGNN^[^ ^19^ ^]^ (mp_traj model). Examples of python code for each UMLPs are given in the Supporting Information. All the considered UMLIPs were available within the atomic simulation environment (ASE),^[^ ^63^ ^]^ which is used to run all the calculations.

### Formation Energies

The formation energy of the host material *F* can be defined as

(1) $$F=E-\sum_{i}\mu_{i}$$

where *E* is the total energy and µ_*i*_ is the chemical potential of atom *i*.

The defect formation energy Δ*F*_*d*_ could be defined as the difference between the formation energy of the defective system *F*_*d*_ and the one from the pristine cell *F*_0_, which reads

(2) $$\Delta F_{d}=F_{d}-F_{0}$$

While Equation (2) works for any defective structure, including substitutional defects in Huang et al. dataset, this work mainly focused on vacancies. In this case, the vacancy formation energy Δ*F*_*v*_ can be written directly using total energies

(3) $$\Delta F_{v}=E_{d}+\mu_{A}-E_{0}$$

where *E*_*d*_ and *E*_0_ denote the total energy of the defective and pristine cells, respectively. The accuracy of UMLIPs to predict Δ*F*_*v*_ would depend on how well they could reproduce the three energy terms. Correlation plot of the total energies for the elemental references between DFT results in MP and those predicted using UMLIPs are shown in Figure S13 (Supporting Information).

The chemical potentials must be carefully chosen to reflect the environment the material was in, i.e., the reservoir with which atoms were exchanged. The elemental phase reference was often adopted in the literature due to convenience and due to the consistency with the phase diagrams. This choice of chemical potential was adopted for the high‐throughput calculations of vacancy formation energy (Section 2.2) and when studying convex hulls (Section 2.3). When considering etching in HF (Figures 4, 5, and 6), instead the values previously used in Ref. (9) were adopted. The values were taken from experiments, but shown to yield good predictions for the probability of etching of MAX phases.^[^ ^36^ ^]^ The list was supplemented with the values for lanthanides when available, taking the experimental values from the NBS tables^[^ ^64^ ^]^ and adding the same corrections as in Ref. (9). A complete list of both sets of chemical potentials are presented in Tables S7 and S8 (Supporting Information).

### High‐Throughput Vacancy Calculations

Conventional unit cells of materials were extracted from MP (v2023.11.1)^[^ ^65^ ^]^ using MP API^[^ ^66^ ^]^ and Pymatgen.^[^ ^67^ ^]^ Supercells were constructed by repeating the unit cell from MP until the lattice constants were larger than 10 Å. The total energy *E*_0_ of the pristine supercell was then calculated using MACE, as well as the total energy of the supercell with a vacancy *E*_*d*_ for every site in the unit cell. The vacancy formation energy was then obtained from Equation (3) and saved in a database available at Ref. (68). Note that while the analysis presented in this work focused on materials close to the convex hull (*E*_hull_ < 0.1 eV), the database also contains vacancy formation energies for materials above the convex hull, leading to a total of 130 321 entries. The database also contains the vacancy formation energy using chemical potentials in the elemental phase as well as in HF solution.

The oxidation states were extracted from MP.^[^ ^65^, ^66^ ^]^ The coordination numbers were determined using CrystalNN algorithm in Pymatgen.^[^ ^69^ ^]^

### Defective Materials Below Convex Hull

For VF_2_, CoI_2_, Ce_2_Mn(SeO)_2_ and RbGdS_2_O_9_, supercells were created following the same method as in high‐throughput vacancy calculations, except for CoI_2_, where larger 6x6x3 supercells were used. Vacancies were created by removing atoms with negative Δ*F*_*v*_ at random. For each concentration, 50 supercells containing vacancies were created and the formation energy was computed before and after relaxation.

Convex hulls from MP were obtained using the MP API.^[^ ^66^ ^]^ It is important to note that anion corrections were applied to formation energies in MP.^[^ ^70^ ^]^ Same corrections were also applied here when calculating convex hull from MACE or DFT calculations.

### 2D Materials from Simulated Etching

From the vacancy formation energy database, 2D layers were obtained by removing atoms with negative vacancy formation energy. Resulting layers with 2D dimensionality higher than 0.5 were then isolated and relaxed. The assessment of the dimensionality and isolation of the layers were done using methods already implemented in the ASE.^[^ ^63^, ^71^ ^]^ Relaxation was performed using the BFGS algorithm with forces and energies calculated with MACE. All 8017 resulting layers were saved in a database available at Ref. (68). Note that this database also contains layers etched from parent materials above the convex hull (*E*_hull_ < 0.1 eV), leading to a higher number of structures than discussed in the text.

In the screening for best candidates, the highest etched element formation energy (HEE) as well as the lowest non‐etched element formation energy (LNE) were used. Given a material with a set of vacancy formation energies Δ*F*_*i*_, the HEE is defined as

(4) $$\text{HEE}=\max_{\Delta F_{i}<0}\Delta F_{i}$$

while the LNE is defined as

(5) $$\text{LNE}=\min_{\Delta F_{i}>0}\Delta F_{i}$$

## Conflict of Interest

The authors declare no conflict of interest.

## Supporting information

Supporting Information

## Data Availability

The data that support the findings of this study are openly available in Zenodo at https://doi.org/10.5281/zenodo.15025795, reference number 15025795.

## References

[1] S. Curtarolo , G. L. W. Hart , M. B. Nardelli , N. Mingo , S. Sanvito , O. Levy , Nat. Mater. 2013, 12, 191. 23422720 10.1038/nmat3568

[2] K. Alberi , M. B. Nardelli , A. Zakutayev , L. Mitas , S. Curtarolo , A. Jain , M. Fornari , N. Marzari , I. Takeuchi , M. L. Green , M. Kanatzidis , M. F. Toney , S. Butenko , B. Meredig , S. Lany , U. Kattner , A. Davydov , E. S. Toberer , V. Stevanovic , A. Walsh , N.‐G. Park , A. Aspuru‐Guzik , D. P. Tabor , J. Nelson , J. Murphy , A. Setlur , J. Gregoire , H. Li , R. Xiao , A. Ludwig , et al., J. Phys. D: Appl. Phys. 2018, 52, 013001.

[3] S. Sanvito , C. Oses , J. Xue , A. Tiwari , M. Zic , T. Archer , P. Tozman , M. Venkatesan , M. Coey , S. Curtarolo , Sci. Adv. 2017, 3, 1602241. 10.1126/sciadv.1602241 PMC5392031 28439545

[4] S. Divilov , H. Eckert , D. Hicks , C. Oses , C. Toher , R. Friedrich , M. Esters , M. J. Mehl , A. C. Zettel , Y. Lederer , E. Zurek , J.‐P. Maria , D. W. Brenner , X. Campilongo , S. Filipović , W. G. Fahrenholtz , C. J. Ryan , C. M. DeSalle , R. J. Crealese , D. E. Wolfe , A. Calzolari , S. Curtarolo , Nature 2024, 625, 66. 38172364 10.1038/s41586-023-06786-y PMC10764291

[5] T. Deng , P. Qiu , T. Yin , Z. Li , J. Yang , T. Wei , X. Shi , Adv. Mater. 2024, 36, 2311278. 10.1002/adma.202311278 38176395

[6] J. Riebesell , T. W. Surta , R. Goodall , M. Gaultois , A. A. Lee , Pushing the pareto front of band gap and permittivity: Ml‐guided search for dielectric materials , 2024, https://arxiv.org/abs/2401.05848.

[7] H.‐C. Wang , S. Botti , M. A. L. Marques , npj Comput. Mater. 2021, 7, 12.

[8] A. R. Oganov , C. J. Pickard , Q. Zhu , R. J. Needs , Nat. Rev. Mater. 2019, 4, 331.

[9] J. Björk , J. Zhou , P. O. A. Persson , J. Rosen , Science 2024, 383, 1210. 38484064 10.1126/science.adj6556

[10] A. Merchant , S. Batzner , S. S. Schoenholz , M. Aykol , G. Cheon , E. D. Cubuk , Nature 2023, 624, 80. 38030720 10.1038/s41586-023-06735-9 PMC10700131

[11] C. Zeni , R. Pinsler , D. Zügner , A. Fowler , M. Horton , X. Fu , Z. Wang , A. Shysheya , J. Crabbé , S. Ueda , R. Sordillo , L. Sun , J. Smith , B. Nguyen , H. Schulz , S. Lewis , C.‐W. Huang , Z. Lu , Y. Zhou , H. Yang , H. Hao , J. Li , C. Yang , W. Li , R. Tomioka , T. Xie , Nature 2025, 639, 624. 39821164 10.1038/s41586-025-08628-5 PMC11922738

[12] V. L. Deringer , M. A. Caro , G. Csányi , Adv. Mater. 2019, 31, 1902765. 10.1002/adma.201902765 31486179

[13] G. Wang , C. Wang , X. Zhang , Z. Li , J. Zhou , Z. Sun , iScience 2024, 27, 109673. 38646181 10.1016/j.isci.2024.109673 PMC11033164

[14] A. Jain , S. P. Ong , G. Hautier , W. Chen , W. D. Richards , S. Dacek , S. Cholia , D. Gunter , D. Skinner , G. Ceder , K. A. Persson , APL Mater. 2013, 1, 011002.

[15] J. Schmidt , N. Hoffmann , H.‐C. Wang , P. Borlido , P. J. M. A. Carriço , T. F. T. Cerqueira , S. Botti , M. A. L. Marques , Adv. Mater. 2023, 35, 2210788. 10.1002/adma.202210788 36949007

[16] R. Tran , J. Lan , M. Shuaibi , B. M. Wood , S. Goyal , A. Das , J. Heras‐Domingo , A. Kolluru , A. Rizvi , N. Shoghi , A. Sriram , F. Therrien , J. Abed , O. Voznyy , E. H. Sargent , Z. Ulissi , C. L. Zitnick , ACS Catalysis 2023, 13, 3066.

[17] C. Chen , S. P. Ong , Nat. Comput. Sci. 2022, 2, 718. 38177366 10.1038/s43588-022-00349-3

[18] B. Deng , P. Zhong , K. Jun , J. Riebesell , K. Han , C. J. Bartel , G. Ceder , Nat. Mach. Intell. 2023, 5, 1031.

[19] K. Choudhary , B. DeCost , L. Major , K. Butler , J. Thiyagalingam , F. Tavazza , Digital Discovery 2023, 2, 346.

[20] I. Batatia , D. P. Kovacs , G. N. C. Simm , C. Ortner , G. Csanyi , in *Adv. Neural. Inf. Process. Syst*. , (Eds.: A. H. Oh , A. Agarwal , D. Belgrave , K. Cho ), 2022, https://openreview.net/forum?id=YPpSngE‐ZU.

[21] I. Batatia , P. Benner , Y. Chiang , A. M. Elena , D. P. Kovács , J. Riebesell , X. R. Advincula , M. Asta , W. J. Baldwin , N. Bernstein , A. Bhowmik , S. M. Blau , V. Cărare , J. P. Darby , S. De , F. D. Pia , V. L. Deringer , R. Elijošius , Z. El‐Machachi , E. Fako , A. C. Ferrari , A. Genreith‐Schriever , J. George , R. E. A. Goodall , C. P. Grey , S. Han , W. Handley , H. H. Heenen , K. Hermansson , C. Holm , et al., 2023, https://arxiv.org/abs/2401.00096.

[22] H. Yang , C. Hu , Y. Zhou , X. Liu , Y. Shi , J. Li , G. Li , Z. Chen , S. Chen , C. Zeni , M. Horton , R. Pinsler , A. Fowler , D. Zügner , T. Xie , J. Smith , L. Sun , Q. Wang , L. Kong , C. Liu , H. Hao , Z. Lu , Mattersim: A deep learning atomistic model across elements, temperatures and pressures, 2024, https://arxiv.org/abs/2405.04967.

[23] Y.‐L. Liao , B. M. Wood , A. Das , T. Smidt , in The Twelfth International Conference on Learning Representations , 2024, https://openreview.net/forum?id=mCOBKZmrzD.

[24] M. Neumann , J. Gin , B. Rhodes , S. Bennett , Z. Li , H. Choubisa , A. Hussey , J. Godwin , Orb: A fast, scalable neural network potential , 2024, https://arxiv.org/abs/2410.22570.

[25] Y. Park , J. Kim , S. Hwang , S. Han , J. Chem. Theory Comput. 2024, 20, 4857. 38813770 10.1021/acs.jctc.4c00190

[26] J. Riebesell , R. E. A. Goodall , P. Benner , Y. Chiang , B. Deng , G. Ceder , M. Asta , A. A. Lee , A. Jain , K. A. Persson , Nat. Mach. Intell. 2025, 7, 836.

[27] T. Angsten , T. Mayeshiba , H. Wu , D. Morgan , New J. Phys. 2014, 16, 015018.

[28] P. Huang , R. Lukin , M. Faleev , N. Kazeev , A. R. Al‐Maeeni , D. V. Andreeva , A. Ustyuzhanin , A. Tormasov , A. H. Castro Neto , K. S. Novoselov , npj 2D Mater. Appl. 2023, 7, 6.

[29] J. Davidsson , F. F. Bertoldo , K. S. Thygesen , R. Armiento , Impurities in 2D Materials Database , 2022, https://data.dtu.dk/articles/dataset/Interstitial_and_Adsorbate_Structure_Database/19692238.

[30] M. H. Rahman , P. Gollapalli , P. Manganaris , S. K. Yadav , G. Pilania , B. DeCost , K. Choudhary , A. Mannodi‐Kanakkithodi , APL Mach. Learn. 2024, 2, 016122.

[31] Y. Kumagai , N. Tsunoda , A. Takahashi , F. Oba , Phys. Rev. Mater. 2021, 5, 123803.

[32] M. D. Witman , A. Goyal , T. Ogitsu , A. H. McDaniel , S. Lany , Nat. Comput. Sci. 2023, 3, 675. 38177319 10.1038/s43588-023-00495-2

[33] V. Ivanov , A. Ivanov , J. Simoni , P. Parajuli , B. Kanté , T. Schenkel , L. Tan , Database of semiconductor point‐defect properties for applications in quantum technologies , 2023, https://arxiv.org/abs/2303.16283.

[34] C. Freysoldt , B. Grabowski , T. Hickel , J. Neugebauer , G. Kresse , A. Janotti , C. G. Van de Walle , Rev. Mod. Phys. 2014, 86, 253.

[35] R. Ibragimova , P. Rinke , H.‐P. Komsa , Chem. Mater. 2022, 34, 2896.

[36] J. Björk , J. Halim , J. Zhou , J. Rosen , npj 2D Mater. Appl. 2023, 7, 5.

[37] J. P. Perdew , K. Burke , M. Ernzerhof , Phys. Rev. Lett. 1996, 77, 3865. 10062328 10.1103/PhysRevLett.77.3865

[38] A. Loew , D. Sun , H.‐C. Wang , S. Botti , M. A. L. Marques , npj Comput. Mater 2025, 11, 178.

[39] H. Yu , M. Giantomassi , G. Materzanini , J. Wang , G.‐M. Rignanese , Mater. Genome Eng. Adv. 2024, 2, e58.

[40] J. Davidsson , F. Bertoldo , K. S. Thygesen , R. Armiento , npj 2D Mater. Appl. 2023, 7, 26.

[41] A. M. Deml , A. M. Holder , R. P. O'Hayre , C. B. Musgrave , V. Stevanović , J. Phys. Chem. Lett. 2015, 6, 1948. 26263275 10.1021/acs.jpclett.5b00710

[42] R. B. Wexler , G. S. Gautam , E. B. Stechel , E. A. Carter , J. Am. Chem. Soc. 2021, 143, 13212. 34428909 10.1021/jacs.1c05570

[43] N. C. Frey , D. Akinwande , D. Jariwala , V. B. Shenoy , ACS Nano 2020, 14, 13406. 32897682 10.1021/acsnano.0c05267

[44] K. Choudhary , B. G. Sumpter , AIP Adv. 2023, 13, 095109.

[45] A. M. Ganose , A. Jain , MRS Commun. 2019, 9, 874.

[46] M. N. Gjerding , A. Taghizadeh , A. Rasmussen , S. Ali , F. Bertoldo , T. Deilmann , N. R. Knøsgaard , M. Kruse , A. H. Larsen , S. Manti , T. G. Pedersen , U. Petralanda , T. Skovhus , M. K. Svendsen , J. J. Mortensen , T. Olsen , K. S. Thygesen , 2D Materials 2021, 8, 044002.

[47] B. Anasori , Y. Gogotsi , Graphene 2D Mater. 2022, 7, 75.

[48] S. Manzeli , D. Ovchinnikov , D. Pasquier , O. V. Yazyev , A. Kis , Nat. Rev. Mater. 2017, 2, 17033.

[49] X. Xu , X. Wang , S. Yu , C. Wang , G. Liu , H. Li , J. Yang , J. Li , T. Sun , X. Hai , L. Li , X. Liu , Y. Zhang , W. Zhang , Q. Zhang , K. Wang , N. Xu , Y. Ma , F. Ming , P. Cui , J. Lu , Z. Zhang , X. Xiao , ACS Nano 2024, 18, 32635. 39530547 10.1021/acsnano.4c10085

[50] G. Villalpando , M. Jovanovic , B. Hoff , Y. Jiang , R. Singha , F. Yuan , H. Hu , D. Călugăru , N. Mathur , J. F. Khoury , S. Dulovic , B. Singh , V. M. Plisson , C. J. Pollak , J. M. Moya , K. S. Burch , B. A. Bernevig , L. M. Schoop , Sci. Adv. 2024, 10, eadl1103. 39303043 10.1126/sciadv.adl1103 PMC11414731

[51] J. Bae , J. Won , T. Kim , S. Choi , H. Kim , S.‐H. V. Oh , G. Lee , E. Lee , S. Jeon , M. Kim , H. W. Do , D. Seo , S. Kim , Y. Cho , H. Kang , B. Kim , H. Choi , J. Han , T. Kim , N. Nemati , C. Park , K. Lee , H. Moon , J. Kim , H. Lee , D. W. Davies , D. Kim , S. Kang , B.‐K. Yu , J. Kim , et al., Nat. Mater. 2024, 23, 1402. 39198713 10.1038/s41563-024-01986-x

[52] P. Hu , Z. Wen , L. Wang , P. Tan , K. Xiao , ACS Nano 2012, 6, 5988. 22676041 10.1021/nn300889c

[53] T. Cao , Z. Li , S. G. Louie , Phys. Rev. Lett. 2015, 114, 236602. 26196815 10.1103/PhysRevLett.114.236602

[54] A. D. Oyedele , S. Yang , L. Liang , A. A. Puretzky , K. Wang , J. Zhang , P. Yu , P. R. Pudasaini , A. W. Ghosh , Z. Liu , C. M. Rouleau , B. G. Sumpter , M. F. Chisholm , W. Zhou , P. D. Rack , D. B. Geohegan , K. Xiao , J. Am. Chem. Soc. 2017, 139, 14090. 28873294 10.1021/jacs.7b04865

[55] E. Chen , W. Xu , J. Chen , J. Warner , Mater. Today Adv. 2020, 7, 100076.

[56] J. F. Sierra , J. Světlík , W. Savero Torres , L. Camosi , F. Herling , T. Guillet , K. Xu , J. S. Reparaz , V. Marinova , D. Dimitrov , S. O. Valenzuela , Nat. Mater. 2025, 24, 876. 39920274 10.1038/s41563-024-02109-2

[57] B. Basu , G. B. Raju , A. K. Suri , Int. Mater. Rev. 2006, 51, 352.

[58] R. L. Davidovich , D. V. Marinin , V. Stavila , K. H. Whitmire , Coord. Chem. Rev. 2015, 299, 61.

[59] V. Kamysbayev , A. S. Filatov , H. Hu , X. Rui , F. Lagunas , D. Wang , R. F. Klie , D. V. Talapin , Science 2020, 369, 979. 32616671 10.1126/science.aba8311

[60] A. Majed , M. Kothakonda , F. Wang , E. N. Tseng , K. Prenger , X. Zhang , P. O. A. Persson , J. Wei , J. Sun , M. Naguib , Adv. Mater. 2022, 34, 2200574. 10.1002/adma.202200574 35419882

[61] M. Rostami Osanloo , A. Saadat , M. L. Van de Put , A. Laturia , W. G. Vandenberghe , Nanoscale 2022, 14, 157. 10.1039/d1nr05250k 34904618

[62] F. Feng , H. Guo , D. Li , C. Wu , J. Wu , W. Zhang , S. Fan , Y. Yang , X. Wu , J. Yang , B. Ye , Y. Xie , ACS Nano 2015, 9, 1683. 25594337 10.1021/nn506473m

[63] A. H. Larsen , J. J. Mortensen , J. Blomqvist , I. E. Castelli , R. Christensen , M. Dułak , J. Friis , M. N. Groves , B. Hammer , C. Hargus , E. D. Hermes , P. C. Jennings , P. B. Jensen , J. Kermode , J. R. Kitchin , E. L. Kolsbjerg , J. Kubal , K. Kaasbjerg , S. Lysgaard , J. B. Maronsson , T. Maxson , T. Olsen , L. Pastewka , A. Peterson , C. Rostgaard , J. Schiøtz , O. Schütt , M. Strange , K. S. Thygesen , T. Vegge , et al., J. Phys.: Condens. Matter 2017, 29, 273002. 28323250 10.1088/1361-648X/aa680e

[64] D. D. Wagman , W. H. Evans , V. B. Parker , R. H. Schumm , I. Halow , S. M. Bailey , K. L. Churney , R. L. Nuttall , J. Phys. Chem. Ref. Data 1982, 11, 2.

[65] A. Jain , S. P. Ong , G. Hautier , W. Chen , W. D. Richards , S. Dacek , S. Cholia , D. Gunter , D. Skinner , G. Ceder , K. a. Persson , APL Mater. 2013, 1, 011002.

[66] S. P. Ong , S. Cholia , A. Jain , M. Brafman , D. Gunter , G. Ceder , K. A. Persson , Comput. Mater. Sci. 2015, 97, 209.

[67] S. P. Ong , W. D. Richards , A. Jain , G. Hautier , M. Kocher , S. Cholia , D. Gunter , V. L. Chevrier , K. A. Persson , G. Ceder , Comput. Mater. Sci. 2013, 68, 314.

[68] The databases presented in this work are available at 10.5281/zenodo.15025795.

[69] H. Pan , A. M. Ganose , M. Horton , M. Aykol , K. A. Persson , N. E. R. Zimmermann , A. Jain , Inorg. Chem. 2021, 60, 1590. 33417450 10.1021/acs.inorgchem.0c02996

[70] A. Wang , R. Kingsbury , M. McDermott , M. Horton , A. Jain , S. P. Ong , S. Dwaraknath , K. A. Persson , Sci. Rep. 2021, 11, 15496. 34326361 10.1038/s41598-021-94550-5 PMC8322326

[71] P. M. Larsen , M. Pandey , M. Strange , K. W. Jacobsen , Phys. Rev. Mater. 2019, 3, 034003.

