# Supplementary material for: Screening of Material Defects using Universal Machine‐Learning Interatomic Potentials
Source: Small. 2025 Aug 3;21(37):e03956. doi: 10.1002/smll.202503956 (PMC12444831; doi:10.1002/smll.202503956)
Supplement: Supplementary file 1 — Supporting Information [file SMLL-21-e03956-s001.pdf]

NANO · MICRO
small

Supporting Information

for *Small*, DOI 10.1002/smll.202503956

Screening of Material Defects using Universal Machine-Learning Interatomic Potentials

*Ethan Berger, Mohammad Bagheri and Hannu-Pekka Komsa**

Supporting Information:
Screening of material defects using universal machine-learning interatomic potentials

Ethan Berger,[1,2] Mohammad Bagheri,[3] and Hannu-Pekka Komsa[1]

[1]*Microelectronics Research Unit, Faculty of Information Technology and Electrical Engineering, University of Oulu, P.O. Box 4500, Oulu, FIN-90014, Finland*
[2]*Department of Physics, Chalmers University of Technology, SE-41296 Gothenburg, Sweden*
[3]*Nanoscience Center, Department of Physics, University of Jyväskylä, Finland*

I. BENCHMARK OF UMLPS

A. Angsten *et al.*

This dataset contains FCC and HCC structures for most of the periodic table. Outputs of the DFT calculations including defects are directly available at Ref. 1, making the comparison straightforward. Reference vacancy formations energies are directly obtained using the energy in the output file, while the defective structures are used to compute the vacancy formation energy with UMLPs. Results for all four UMLPs are shown in Fig. S1.

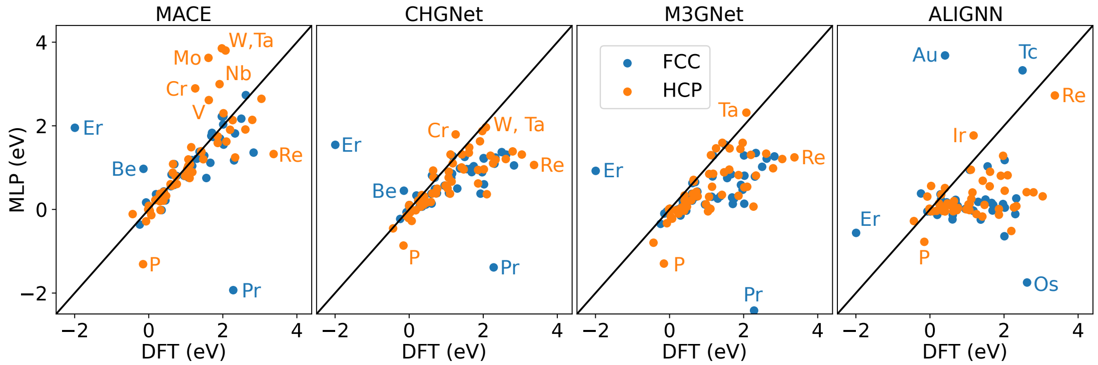

FIG. S1. Comparison of the defect formation energy from DFT calculations with (a) MACE, (b) CHGNet, (c) M3GNet and (d) ALIGNN. Reference DFT values are taken from Angsten *et al*. [2].

B. Huang *et al.*

Dataset from Ref. 3 contains defect formation energies for 6 different 2D layers with high density of defects (vacancies and substitutions). Note however that only the initial unrelaxed defective structures are shared. These are therefore first relaxed using MACE, and the defect formation energy of the resulting relaxed structures is then computed using all four UMLPs. Results are shown in Fig. S2. Note that the good agreement between MACE and DFT proves that structures were correctly relaxed.

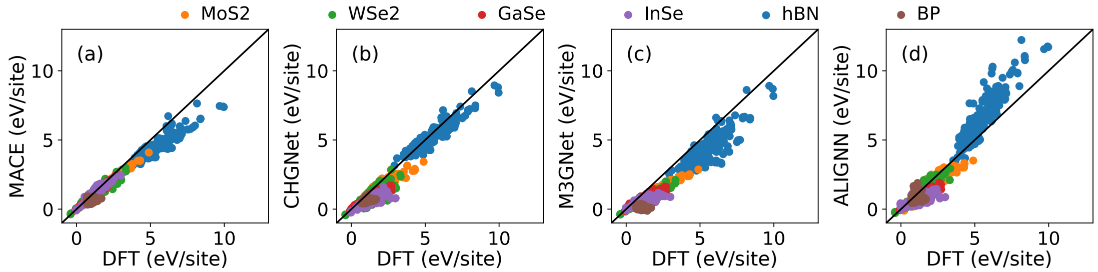

FIG. S2. Comparison of the defect formation energy from DFT calculations with (a) MACE, (b) CHGNet, (c) M3GNet and (d) ALIGNN. Reference DFT values are taken from Huang *et al*.[3].

C. Björk *et al.*

In Ref. 4, Björk *et al*. screened the Materials Project database in a similar way to what is presented in the main text. As a result, they obtain a database with defect formation energies for every sites of many materials, which can be grouped into three main families : TMDCs, MXenes and RuSi-like. For TMDCs, we used either $LiNbS_2$ or $InNbS_2$ as a starting layer, depending if the A sites were alkali metals or post-transision metals. Other structures were then created by substitution of the elements and relaxation using MACE. Similarly, for MXenes and RuSi-like, we started with $Ti_{n+1}AlC_n$ and YRu_2Si_2 and performed the same substitution and relaxation to obtain the rest of the structures. Defect formation energies were then calculated using the final relaxed structures and all four UMLPs. A comparison between DFT results and UMLPs predictions is shown in Fig. S3. Here again, note that the good agreement between MACE and DFT proves that structures were created correctly.

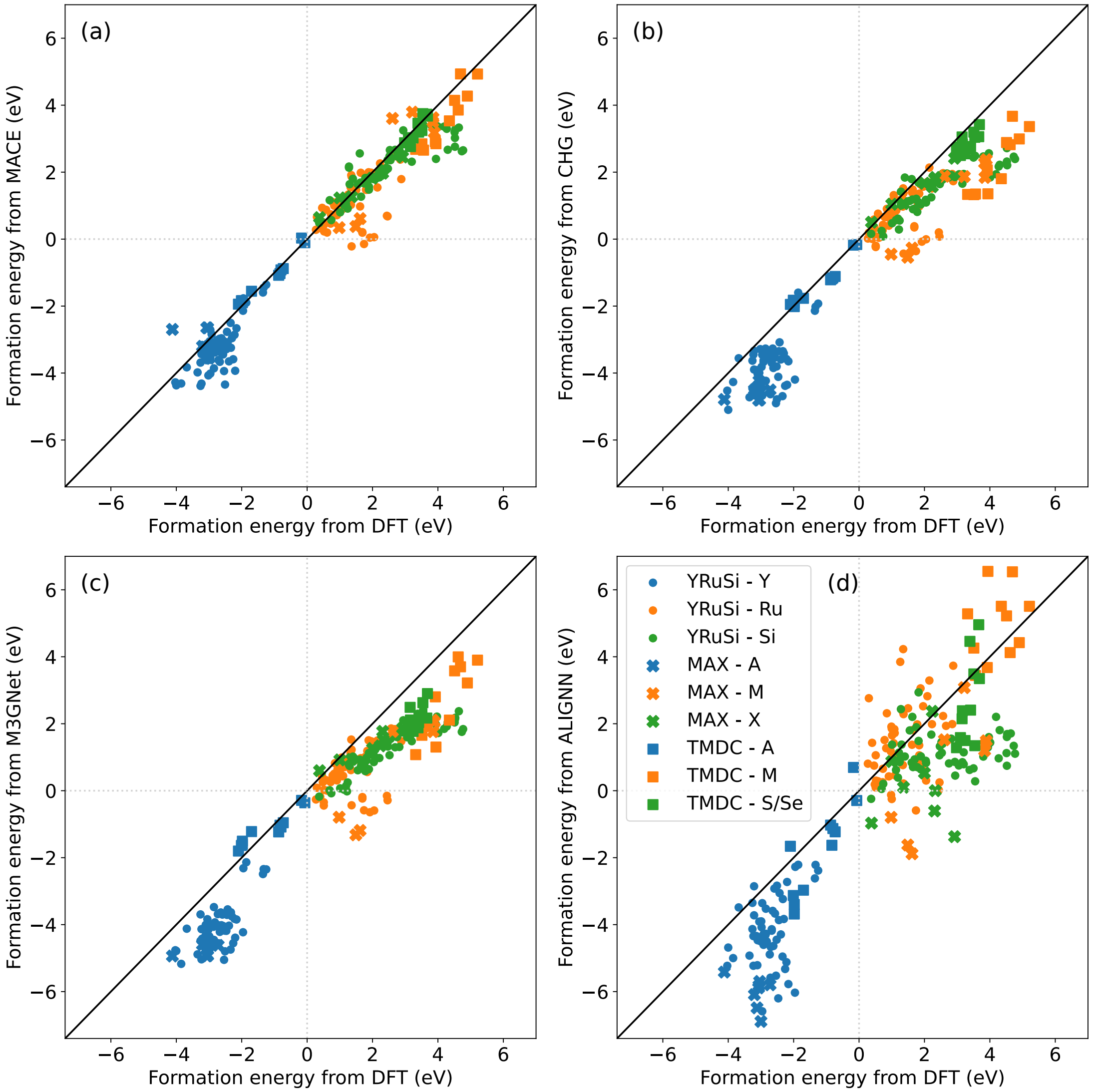

FIG. S3. Comparison of the defect formation energy from DFT calculations with (a) MACE, (b) CHGNet, (c) M3GNet and (d) ALIGNN. Reference DFT values are taken from Björk *et al*. [4].

D. Other datasets

In Fig. S4 we show the results from the defect database of Davidsson *et al.* [5]. It contains interstitial and adatom impurities on 2D materials. Finally, in Fig. S5 we show the results from the defect database of Choudhary *et al.* [6], which covers vacancy, substitutional, and interstitial defects in few common semiconductors and insulators. We think that the downshift of the UMLP values might arise from the adopted chemical potentials, for which we could not find proper documentation.

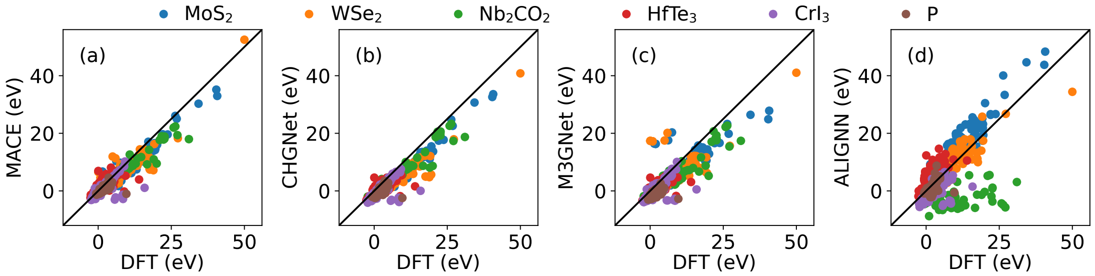

FIG. S4. Comparison of the defect formation energy from DFT calculations with (a) MACE, (b) CHGNet, (c) M3GNet and (d) ALIGNN. Reference DFT values are taken from Ref. 5.

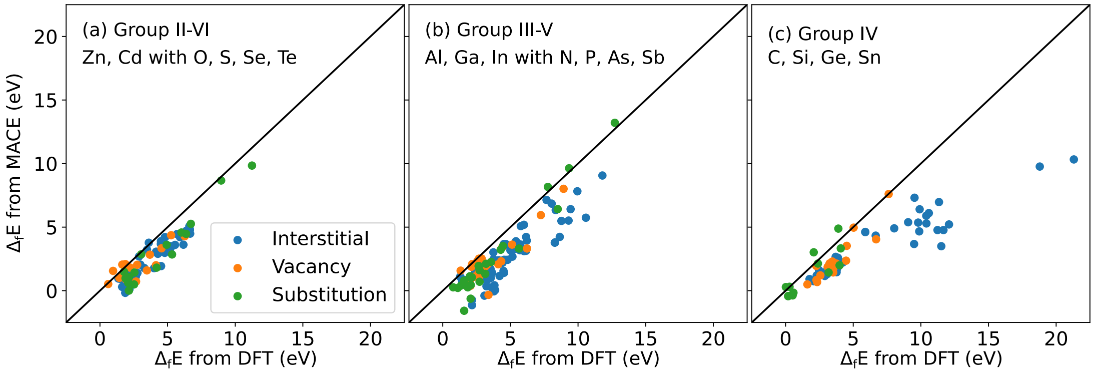

FIG. S5. Comparison of the defect formation energy from DFT calculations with MACE in various semiconductors. Different types of defects are separated by colors. Reference DFT values are taken from Ref. 6.

A summary of the RMSE of the four UMLIPs over the five datasets is presented below.

Dataset	MACE	M3GNet	CHGNet	ALIGNN
Angsten *et al.* [2]	0.80	1.01	0.85	5.31
Huang *et al.* [3]	0.46	0.82	0.49	0.60
Björk *et al.* [4]	0.67	1.12	1.34	1.69
Davidsson *et al.* [5]	2.34	3.09	3.74	5.16
Choudhary *et al.* [6]	2.19	9.82	2.99	38.34

TABLE S1. RMSE of the UMLIPs for the all benchmarked datasets. All values are in eV.

II. OXIDATION STATES AND COORDINATION NUMBER

Fig. 2(d) and (e) of the main text show the distribution of vacancy formation energy for different oxidation states of various elements. The oxidation states were obtained using pymatgen and their distribution is presented in Table S2. For some elements, there are many possible oxidation states. In Fig. 2(d-e), we only considered oxidation states with ratio higher than 20%, which are shown in bold in the following tables.

	-4	-3	-2	-1	0	1	2	3	4	5	6	7
H				14.8	0.4	**84.8**						
Li					0.1	**99.9**						
Be							**100.0**					
B		5.2	12.5	8.8	0.4	2.7	1.7	**68.7**				
C	**29.5**	11.3	13.7	2.7	0.4		1.6	2.0	**39.0**			
N		**86.4**	1.5	0.6	0.2	0.3	0.1	0.3		10.6		
O			**99.8**	0.1	0.0	0.0	0.0	0.0	0.0	0.0		
F				**100.0**					0.0			
Na						**99.9**	0.0	0.1				
Mg					0.1		**99.9**					
Al							0.1	**99.9**				
Si	**21.6**	0.1	1.3	3.1			0.2	0.6	**73.2**			
P		14.5	2.3	2.2	0.9	0.8	1.2	1.0	4.2	**73.2**		
S			**80.6**	5.6	0.4	0.1	0.2	0.0	0.3	0.1	12.7	
Cl				**98.8**				0.2		0.3		0.7
K			0.3		0.2	**99.4**				0.1		
Ca					0.2		**99.8**	0.0				
Sc					0.1	18.2	12.0	**69.7**				
Ti					0.2		6.2	11.6	**82.1**			
V							3.8	**24.6**	**24.0**	**47.6**		
Cr					0.1		10.0	**55.5**	7.5	10.3	16.6	
Mn						1.3	**53.0**	**25.4**	18.2	1.3	0.5	0.3
Fe					0.0	7.7	**37.4**	**47.4**	4.2	2.1	1.0	
Co					0.1	**33.4**	**45.8**	13.1	7.7			
Ni						**27.9**	**56.7**	10.2	5.1			
Cu			0.1		0.1	**55.2**	**39.8**	4.7				
Zn					0.1		**99.9**					
Ga						2.3	6.9	**90.8**				
Ge	**22.5**		16.5				4.0	2.4	**54.6**			
Se			**68.3**	12.7	1.1	0.3	0.0		12.9	0.2	4.5	
Br				**97.9**		0.1	0.4	0.4		1.2		
Rb			0.1		0.6	**99.2**		0.1				
Sr			0.3		0.0		**99.7**					
Y			0.2		0.3	3.0	0.2	**96.3**				
Zr				0.5	0.1	14.2	11.9	11.9	**61.4**			
Nb			0.4			5.4	12.4	16.5	6.2	**59.0**		0.1
Mo							12.3	5.4	7.1	13.9	**61.3**	
Ru					0.2		**23.4**	9.0	19.2	**42.0**	6.1	
Rh						**43.1**		**38.4**	18.5			
Pd							**79.5**	13.3	7.2			
Ag					0.3	**93.3**	4.5	1.9				
Cd					0.1		**99.9**					
In					0.1	15.7	10.6	**73.6**				
Sn							**41.4**	2.4	**56.3**			
Sb		**26.6**	19.3	7.1	0.3			**29.2**	0.2	17.3		
Te			**50.8**	14.9	0.1	0.6	0.2		**27.3**	0.3	5.7	
I				**89.2**		0.6		0.3		9.0		0.9
Cs						**100.0**						
Ba							**100.0**					
La					0.1	3.3	6.2	**90.4**				
Ce			0.3				15.1	**65.5**	19.2			
Pr					0.6		4.6	**93.3**	1.5			
Nd					0.2		6.9	**92.9**				
Sm					0.2		9.0	**90.8**				
Eu			1.2			1.2	**62.2**	**35.4**				

	-4	-3	-2	-1	0	1	2	3	4	5	6	7
Gd					0.2		2.2	**97.6**				
Tb					0.4	10.3	12.5	**68.2**	8.7			
Dy					0.4		8.7	**90.9**				
Ho					0.3		10.1	**89.6**				
Er					0.3			**99.7**				
Tm					0.2		10.2	**89.6**				
Lu					0.2			**99.8**				
Hf					0.2		8.0	1.3	**90.6**			
Ta					2.1	11.8	8.5	6.7	6.8	**64.1**		
W							14.2	4.9	4.9	11.2	**64.8**	
Re					0.5		1.2	**30.7**	7.8	18.2	10.6	**31.1**
Os					1.7				**98.3**			
Ir								**31.1**	**28.5**	**33.9**	6.6	
Pt							**44.8**	9.7	**45.6**			
Au			2.0					**98.0**				
Hg					2.3	16.2	**81.5**					
Tl					0.2	**87.7**		12.0				
Pb							**95.9**	0.9	3.2			
Bi		6.3			0.3	1.5	2.6	**80.5**	1.2	7.6		

TABLE S2: Distribution of the oxidation states for elements from Cs to Bi. Oxidation states with ratio over 20% are shown in bold.

The average vacancy formation energies of element at given oxidation state are listed in Table S3.

	-4	-3	-2	-1	0	1	2	3	4	5	6	7
H				1.1	2.1	**2.7**						
Li					0.6	**3.6**						
Be							**8.5**					
B		1.7	2.1	2.2	2.5	3.9	4.1	**9.7**				
C	**2.1**	1.4	2.0	2.9	6.9		5.5	7.5	**8.5**			
N		**3.7**	4.1	3.5	5.5	3.6	-2.0	4.0		7.2		
O			**4.4**	1.5	2.7	3.0	4.8	1.9	3.6	4.9		
F				**3.8**					3.2			
Na						**3.1**	3.7	4.4				
Mg					0.8		**6.8**					
Al							1.9	**10.1**				
Si	**2.5**	2.1	1.5	0.9			2.2	2.5	**12.7**			
P		2.7	1.9	1.5	1.0	2.9	1.6	3.5	4.4	**13.7**		
S			**2.3**	1.1	1.2	1.9	2.9	2.2	7.1	11.6	13.4	
Cl				**2.2**				3.8		6.1		8.3
K			2.2		0.2	**3.2**				2.8		
Ca					1.1		**7.1**	9.0				
Sc					1.6	2.5	2.2	**9.7**				
Ti					1.6		2.4	5.1	**12.0**			
V							1.2	**5.7**	**8.0**	**11.2**		
Cr					3.0		1.9	**5.6**	6.4	7.6	10.7	
Mn						0.8	**3.1**	**5.4**	5.9	7.5	8.5	8.1
Fe					1.9	1.2	**1.4**	**4.8**	3.6	2.1	3.3	
Co					1.3	**1.4**	**1.9**	2.8	3.0			
Ni						**1.2**	**1.6**	1.9	1.7			
Cu			0.1		1.0	**0.5**	**2.4**	4.6				
Zn					0.3		**3.9**					
Ga						1.7	2.3	**6.9**				
Ge	**2.4**		2.0				2.1	3.0	**8.8**			
Se			**1.9**	1.1	1.0	1.3	-0.2		7.5	9.1	11.1	
Br				**2.0**		0.2	1.9	4.3		5.3		
Rb			4.2		0.1	**3.1**		4.3				
Sr			7.6		0.8		**6.6**					
Y			6.2		1.8	3.4	7.1	**10.4**				
Zr				21.1	2.3	2.5	3.1	4.0	**12.8**			
Nb			15.1			2.2	2.4	3.3	5.7	**12.5**		15.9
Mo							2.8	3.5	4.1	7.4	**10.7**	

Ru					1.8		**1.9**	2.3	4.9	**7.4**	9.5	
Rh						**1.6**		**2.7**	3.9			
Pd							**1.7**	1.5	3.1			
Ag					0.5	**0.5**	2.6	3.5				
Cd					0.3		**3.0**					
In					0.4	1.1	2.4	**5.6**				
Sn							**3.2**	4.0	**7.1**			
Sb		**2.1**	1.6	1.3	3.0			**4.6**	12.1	12.6		
Te			**2.1**	1.1	0.5	1.1	1.7		**7.6**	9.4	12.8	
I				**1.4**		1.1		3.7		8.1		10.9
Cs						**3.1**						
Ba							**6.4**					
La					1.4	2.9	3.2	**8.9**				
Ce			6.7				2.6	**5.5**	9.9			
Pr					1.5		3.5	**8.3**	7.5			
Nd					1.6		3.9	**9.5**				
Sm					1.5		3.6	**9.3**				
Eu			10.6			12.0	**4.5**	**9.6**				
Gd					1.4		3.1	**10.2**				
Tb					1.7	2.9	2.9	**9.5**	5.9			
Dy					1.8		3.2	**9.1**				
Ho					1.8		3.2	**9.1**				
Er					1.6			**9.3**				
Tm					1.6		3.5	**9.3**				
Lu					1.5			**9.6**				
Hf					2.2		3.9	3.4	**10.0**			
Ta					3.7	2.3	2.2	3.2	4.9	**13.6**		
W							1.7	2.7	3.9	6.3	**9.3**	
Re					1.3		4.3	**3.5**	5.0	3.2	8.6	**15.2**
Os					2.6				**3.0**			
Ir								**2.8**	**4.4**	**5.9**	8.9	
Pt							**2.2**	3.3	**5.7**			
Au			3.5					**3.3**				
Hg					0.0	1.7	**1.9**					
Tl					0.3	**1.4**		5.2				
Pb							**3.8**	5.7	7.7			
Bi		2.2			0.6	1.5	1.6	**5.0**	7.9	10.5		
	-4	-3	-2	-1	0	1	2	3	4	5	6	7

TABLE S3: Distribution of the average vacancy formation energies for elements from Cs to Bi. Oxidation states with ratio over 20% are shown in bold (same as in Table S2).

In addition to the oxidation number, the main text also discusses the impact of the coordination number on the vacancy formation energies. Results can be found in Fig. S9 for transition metals and in Fig. S6 for other elements.

Fig. 2(d) of the main text shows the vacancy formation energy for various oxidation states of 3d transition metals. A complete picture including all transition metal elements is shown in Fig. S7.

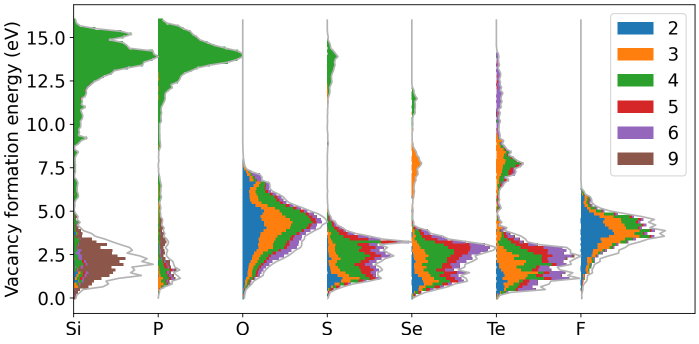

FIG. S6. Vacancy formation energies of various elements with different coordination numbers.

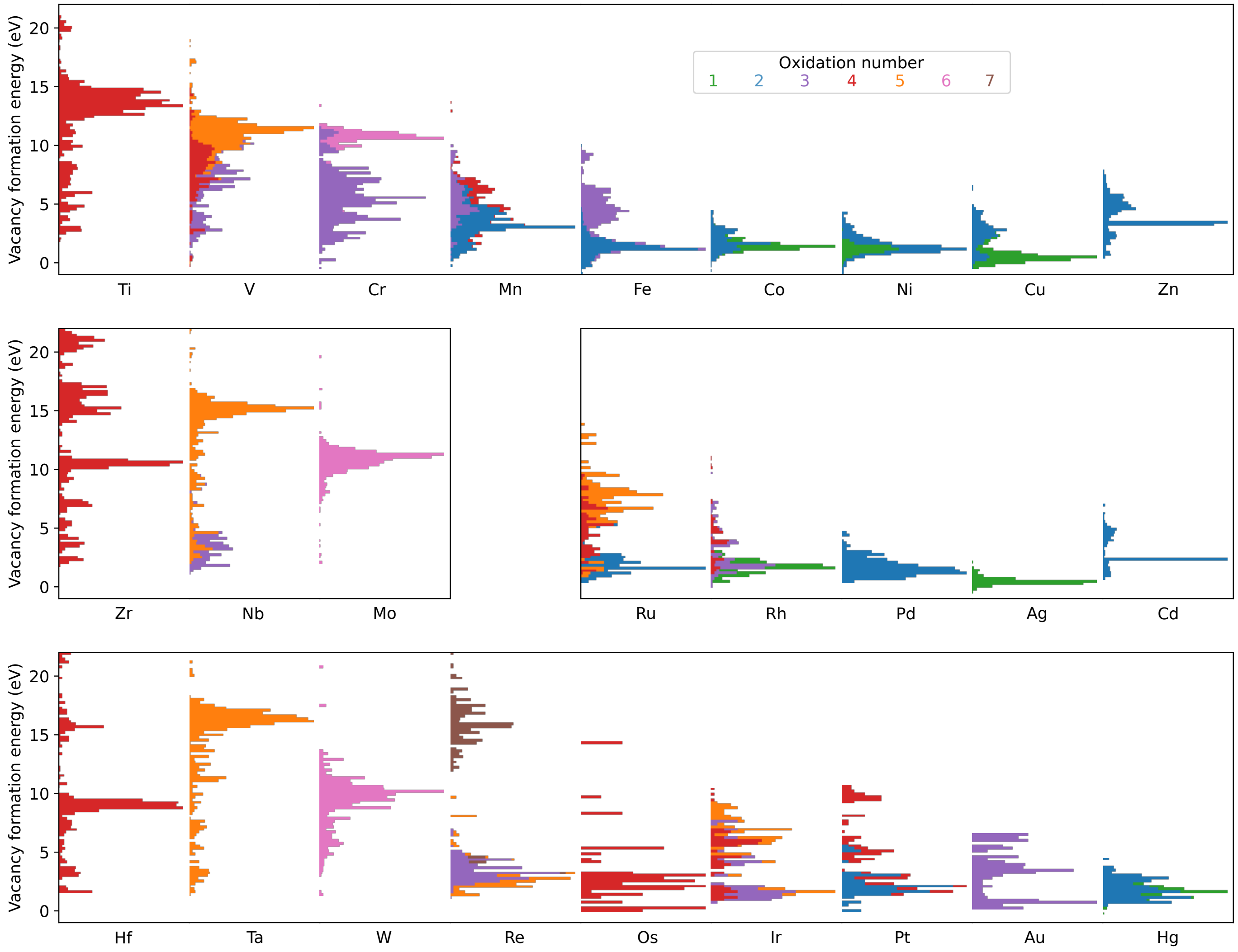

FIG. S7. Stacked histograms of the vacancy formation energies for the 3d, 4d, and 5d transition metals.

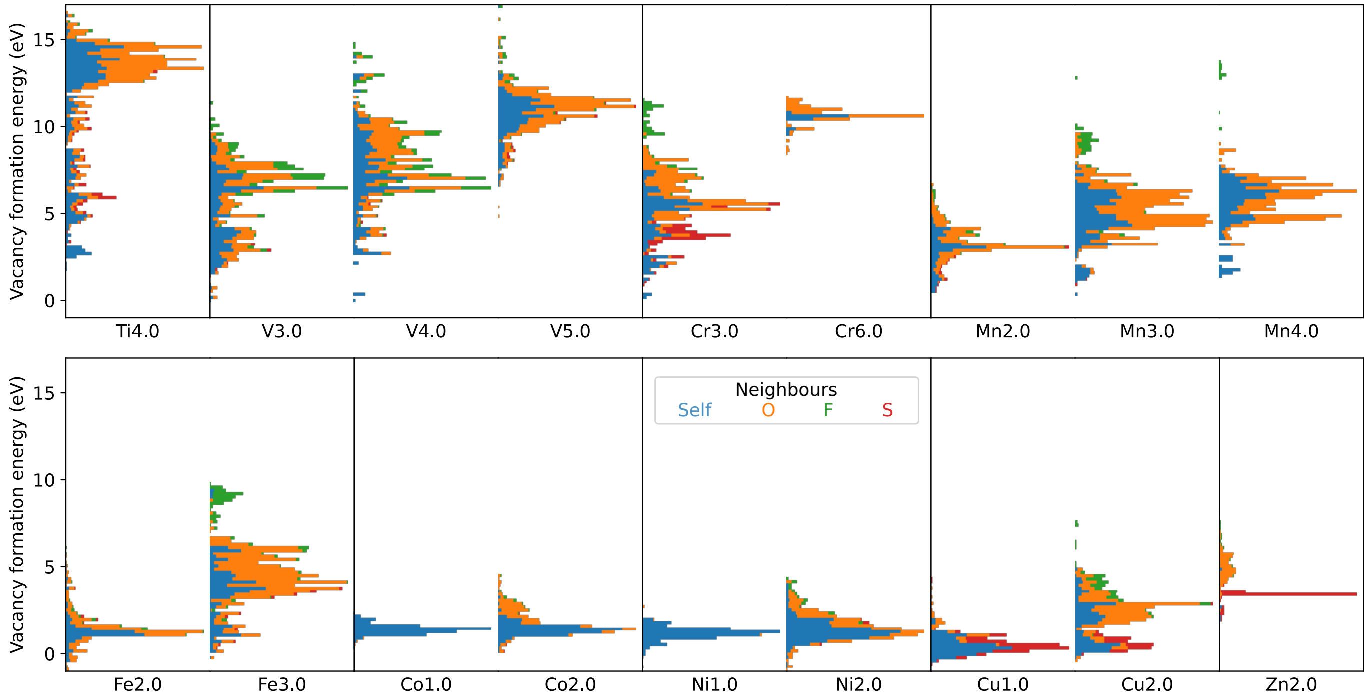

FIG. S8. Stacked histograms of the vacancy formation energies for the 3d transition metals in their most common oxidation states. Each histogram is separated depending on the atoms neighboring the transition metal atom. Note that vacancies with two (or more) neighboring elements would be included in both (or all) corresponding histograms.

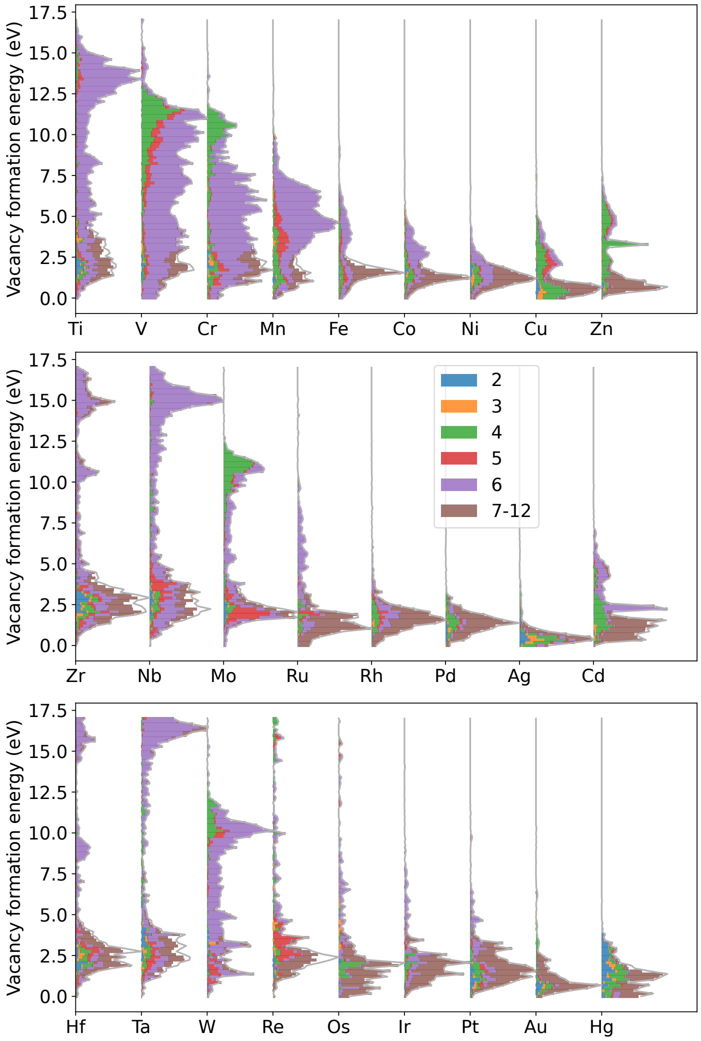

FIG. S9. Vacancy formation energies of transition metals with different coordination numbers.

III. VACANCIES IN BULK MATERIALS

As presented in the main text, the high-throughput calculation of vacancy formation energy highlighted 34 materials with $\Delta F_v < -0.75$. While Fig. 3 of the main text presents in-depth analysis of 4 of these materials, the rest of them are presented below. In particular, Fig. S10 shows the unrelaxed formation energy with increasing vacancy concentration [similar to Fig. 3(a)]. Materials are presented in 5 different panels for clarity: panel (a) shows the materials which can accept a low amount of vacancies (similar to VF_2 in the main text). Panel (b) presents materials similar to CoI_2 and $Ce_2Mn(SeO)_2$ which can accept a larger amount of vacancies and show minimal formation energies at defect concentration between 20 and 50%. Materials with negative formation energies at 100% defect concentration are shown in panels (c), (d) and (e).

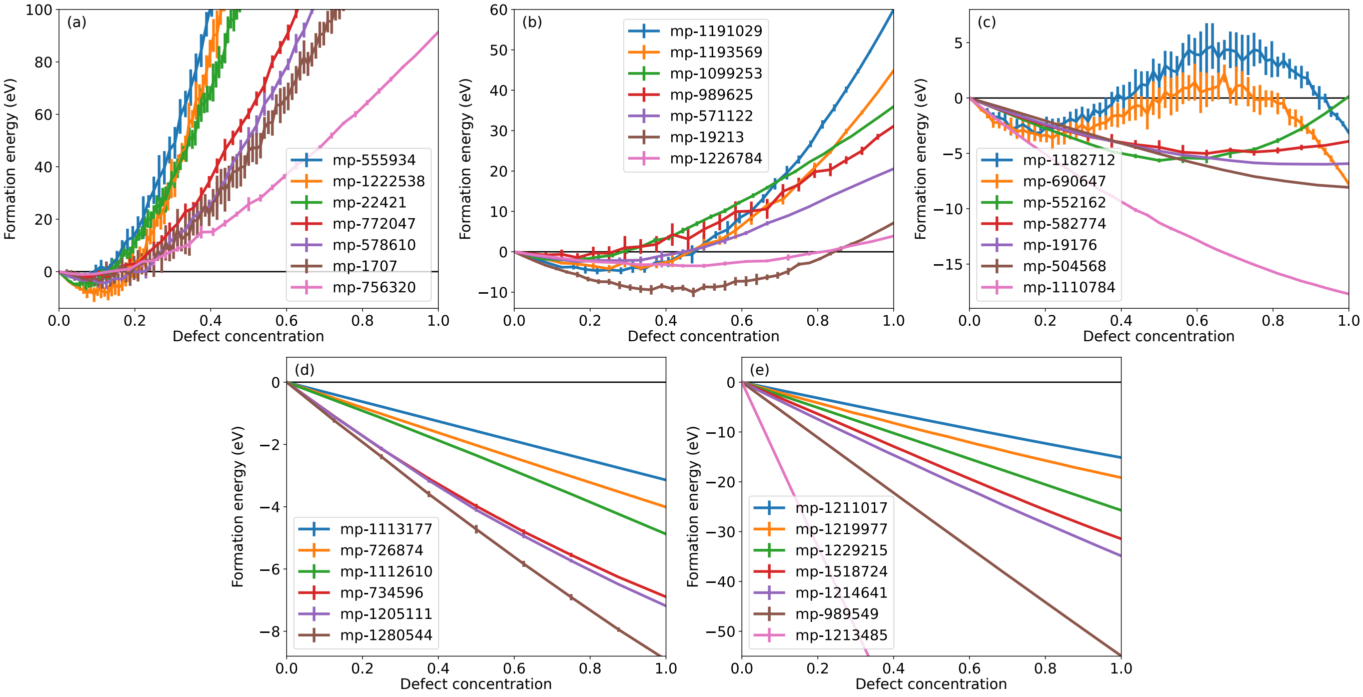

FIG. S10. Unrelaxed defect formation energy with respect to the vacancy concentration for all materials with $\Delta F_v < -0.75$. Curves are separated in categories and plotted in 5 different panels for an easier graphical representation.

Table S4 compiles the MPID, formula, lowest vacancy formation energy and etched atoms for all 34 materials.

In addition to the 4 materials presented in details in the main text, we could mention two other interesting cases. First, $Na_3Mo_3O_6$ (mp-578610) is made of MoO_2 2D layers with intercalated Na ions. The formation energy is minimal after etching of 10-12% of Mo atoms, leading to a molybdenum-oxide layer similar to CoI_3 or Mn_3S_8 [see Figs. 3(f) and 5(c) respectively]. The other interesting case is $CaBr_2O_8(H_2O)_4$ (mp-1182712) and $NiBr_2O_8(H_2O)_6$ (mp-690647). These two materials contain water molecules and BrO_4 tetrahedra, with the oxygen of the tetrahedra having negative vacancy formation energy. The curves in Fig. S10 show two local minima at defect of 25% and 100%, corresponding to the BrO_4 units being respectively replaced by BrO_3 and Br.

MPID	Formula	Lowest ΔF_v (eV)	Etched atoms	Formula after etching
mp-555934	VF_2	-0.812	V	F_2
mp-1222538	LiV_3O_4	-1.446	V_3	LiO_4
mp-22421	Fe_2GeO_4	-1.230	Fe_2	GeO_4
mp-772047	V_2O_4Si	-1.260	V_2	SiO_4
mp-578610	$Na_3Mo_3O_6$	-1.575	Mo_3	Na_3O_6
mp-1707	Ba_2N_{12}	-0.800	N_8	Ba_2N_4
mp-756320	$SbLi_3Fe_4O_8$	-1.085	Fe_4	$SbLi_3O_8$
mp-1191029	$Pr_4C_4O_{16}$	-1.056	O_4	$Pr_4C_4O_{12}$
mp-1193569	$La_4S_4O_{20}$	-1.034	O_4	$La_4S_4O_{16}$
mp-1099253	Mg_3NiO_4	-1.061	Ni	Mg_3O_4
mp-989625	$Sr_2W_2N_6$	-0.758	N_2	$Sr_2W_2N_4$
mp-571122	FeI_2	-0.997	Fe	I_2
mp-19213	$Ba_4Mn_4O_2Sb_4$	-1.188	Mn_4	$Ba_4O_2Sb_4$
mp-1226784	$Ce_2MnO_2Se_2$	-0.804	Mn	$Ce_2O_2Se_2$
mp-1182712	$CaBr_2O_8(H_2O)_4$	-0.788	O_8	$CaBr_2(H_2O)_4$
mp-690647	$NiBr_2O_8(H_2O)_6$	-0.768	O_8	$NiBr_2(H_2O)_6$
mp-552162	$Fe_8Re_8Sr_{16}O_{48}$	-0.868	Fe_8	$Re_8Sr_{16}O_{48}$
mp-582774	$Ce_8Mn_2O_2Se_{12}$	-0.836	Mn_2	$Ce_8O_2Se_{12}$
mp-19176	$La_4Ni_2Ru_2O_{12}$	-0.792	Ni_2	$La_4Ru_2O_{12}$
mp-504568	$Ba_{12}Ni_6Os_6O_{36}$	-0.890	Ni_6	$Ba_{12}Os_6O_{36}$
mp-1110784	$Ag_4Mo_4Rb_8F_{24}$	-0.847	Ag_4	$Mo_4Rb_8F_{24}$
mp-1113177	$Cs_8Hg_4Mo_4F_{24}$	-0.785	Hg_4	$Cs_8Mo_4F_{24}$
mp-726874	$Zn_2C_4N_{16}O_8$	-1.019	Zn_2	$C_4N_{16}O_8$
mp-1112610	$Cs_8Hg_4Ta_4F_{24}$	-1.150	Hg_4	$Cs_8Ta_4F_{24}$
mp-734596	$Cs_4Os_8O_{36}$	-1.090	O_4	$Cs_4Os_8O_{32}$
mp-1205111	$Rb_4Os_8O_{36}$	-1.104	O_4	$Rb_4Os_8O_{32}$
mp-1280544	$Mn_4Ni_2V_6O_{16}$	-1.314	Ni_2	$Mn_4V_6O_{16}$
mp-1211017	$Gd_4Rb_4O_{36}S_8$	-1.996	O_4	$Gd_4Rb_4O_{32}S_8$
mp-1219977	$Cu_3Pr_3Ge_3$	-0.875	Cu_3	Pr_3Ge_3
mp-1229215	$Al_{22}O_{36}$	-3.190	O_2	$Al_{22}O_{34}$
mp-1518724	$Ba_4Cr_4Fe_4Gd_4O_{24}$	-0.973	Fe_4	$Ba_4Cr_4Gd_4O_{24}$
mp-1214641	$Ba_4Re_8O_{36}$	-2.353	O_4	$Ba_4Re_8O_{32}$
mp-989549	$Cs_8Pb_4Cl_4F_{24}$	-1.737	Cl_4	$Cs_8Pb_4F_{24}$
mp-1213485	$Cu_4N_8O_{32}S_8$	-5.185	N_8	$Cu_4O_{32}S_8$

TABLE S4. MPID, formula, lowest formation energy, etched atoms and formula after etching for all 34 bulk materials with vacancy formation energies below -0.75 eV.

IV. BEST CANDIDATES

MP ID	Etched	Layer	HEE	LNE	LDFE	Inital Dim.
mp-1232339	Li	C	-3.28	6.39	6.67	2D : 0.499
mp-1021323	Li	C	-3.3	6.4	6.67	02D : 0.494
mp-1001581	Li	C	-3.19	5.53	6.67	3D : 0.54
mp-28930	K	C	-3.18	5.35	6.67	3D : 0.803
mp-568643	Rb	C	-3.2	5.28	6.67	3D : 0.847
mp-28861	Cs	C	-3.26	5.51	6.67	3D : 0.922
mp-1208630	Sr	C	-5.61	4.07	6.67	3D : 0.907
mp-1214417	Ba	C	-5.57	4.2	6.68	3D : 0.926
mp-1025297	Sm	C	-6.48	4.42	6.67	3D : 1.0
mp-1103990	Eu	C	-4.68	3.71	6.68	3D : 0.984
mp-19755	Li	TiS_2	-1.46	1.39	2.16	2D : 0.742
mp-1223278	Li	TiS_2	-1.21	1.39	2.16	3D : 0.734
mp-675056	Na	TiS_2	-1.04	1.92	2.16	2D : 0.899
mp-1048589	Ca	TiS_2	-1.86	2.08	2.16	2D : 0.96
mp-1048662	Ca	TiS_2	-2.0	1.63	2.16	3D : 1.0
mp-1048666	Ca	TiS_2	-2.11	0.837	2.16	3D : 1.0
mp-1386933	Ca	TiS_2	-1.96	1.33	2.16	3D : 1.0
mp-756195	Li	VS_2	-2.2	1.87	1.45	3D : 1.0
mp-1177739	Li	VS_2	-1.52	1.52	1.39	3D : 0.931
mp-676586	Na	VS_2	-1.09	1.59	1.39	3D : 0.911
mp-675593	K	VS_2	-1.44	1.57	1.55	3D : 0.994
mp-1223993	K	VS_2	-1.37	1.78	1.39	3D : 0.962
mp-1400988	Ca	VS_2	-2.39	1.46	1.6	3D : 1.0
mp-1216498	V	VS_2	-0.893	0.882	1.53	3D : 1.0
mp-4226	Li	CrS_2	-1.05	1.62	1.18	3D : 0.617
mp-755947	Li	CrS_2	-0.935	2.03	1.15	3D : 0.838
mp-1238813	Li	CrS_2	-1.21	1.61	1.15	3D : 0.725
mp-1238817	Li	CrS_2	-0.753	1.67	1.18	3D : 0.674
mp-5693	Na	CrS_2	-1.16	1.87	1.18	3D : 0.93
mp-637292	Na	CrS_2	-1.15	1.88	1.18	3D : 0.905
mp-1238847	Na	CrS_2	-0.869	1.4	1.15	3D : 0.866
mp-4026	K	CrS_2	-1.74	2.02	1.18	3D : 0.997
mp-1238855	K	CrS_2	-1.56	1.37	1.15	3D : 0.971
mp-1238878	Rb	CrS_2	-1.13	1.32	1.15	3D : 0.986
mp-1238845	Cs	CrS_2	-0.878	1.33	1.15	3D : 1.0
mp-1390152	Mg	CrS_2	-1.63	1.57	1.18	3D : 0.917
mp-2227138	Mg	CrS_2	-2.55	1.39	1.18	2D : 0.889
mp-1393946	Ca	CrS_2	-0.965	1.45	1.18	3D : 1.0
mp-1221432	Na	$CrSe_2$	-1.11	1.34	1.39	3D : 0.944
mp-30248	Li	MoS_2	-1.61	2.88	1.66	3D : 0.682
mp-1025173	Ti	MoS_2	-1.97	1.42	1.63	3D : 1.0
mp-555370	V	MoS_2	-1.71	1.78	1.63	3D : 0.999
mp-1192730	V	MoS_2	-1.75	1.76	1.62	3D : 0.999
mp-542188	Cr	MoS_2	-2.06	1.68	1.62	3D : 0.991
mp-1087488	In	MoS_2	-1.71	3.18	2.87	2D : 0.474
mp-1199798	Cs	ReS_2	-1.16	1.73	0.752	3D : 1.0
mp-1077996	Cr	$RhSe_2$	-1.83	1.01	1.01	3D : 1.0
mp-1078162	Fe	$RhSe_2$	-1.35	1.04	1.01	3D : 1.0
mp-1078249	Co	$RhSe_2$	-0.896	0.996	1.01	3D : 0.999
mp-1077939	Cr	$RhTe_2$	-2.29	1.31	1.68	3D : 0.999
mp-7936	Li	NbS_2	-1.56	3.2	1.78	3D : 0.719
mp-767218	Li	NbS_2	-1.14	2.58	1.82	3D : 0.695
mp-7937	Na	NbS_2	-1.8	3.63	1.78	3D : 0.955
mp-1221395	Na	NbS_2	-1.6	2.64	1.78	2D : 0.943
mp-1221460	Na	NbS_2	-1.15	2.84	1.78	3D : 0.906
mp-1224044	K	NbS_2	-1.48	2.89	1.82	3D : 0.991
mp-7938	K	NbS_2	-2.66	3.73	1.82	3D : 1.0
mp-1229211	Cs	NbS_2	-1.41	2.62	1.79	3D : 1.0
mp-1188929	Ti	NbS_2	-0.897	3.18	1.82	3D : 1.0
mp-1189260	Cr	NbS_2	-0.984	2.9	1.82	3D : 1.0

mp-15958	V	NbS_2	-0.815	3.01	1.82	3D : 1.0
mp-10199	Mn	NbS_2	-1.58	2.74	1.78	3D : 0.959
mp-1220734	Fe	NbS_2	-2.16	2.33	1.78	2D : 0.952
mp-20621	In	NbS_2	-1.5	2.87	1.82	02D : 0.614
mp-1018022	In	NbS_2	-0.902	2.88	1.82	3D : 0.546
mp-1025496	Li	$NbSe_2$	-1.92	2.61	2.05	3D : 0.797
mp-7939	Na	$NbSe_2$	-1.94	2.84	2.11	3D : 0.985
mp-1221396	Na	$NbSe_2$	-1.84	2.85	2.05	2D : 0.944
mp-1221482	Na	$NbSe_2$	-1.84	2.84	2.05	2D : 0.913
mp-7940	K	$NbSe_2$	-2.59	2.66	2.11	3D : 1.0
mp-1224047	K	$NbSe_2$	-1.7	2.97	2.11	3D : 1.0
mp-1025291	Ti	$NbSe_2$	-2.17	1.97	2.26	3D : 0.994
mp-1188631	Ti	$NbSe_2$	-2.11	2.47	2.04	3D : 1.0
mp-1025195	V	$NbSe_2$	-2.19	2.19	2.26	3D : 0.99
mp-1105840	V	$NbSe_2$	-2.08	2.66	2.04	3D : 1.0
mp-1209978	V	$NbSe_2$	-1.75	2.84	2.04	3D : 1.0
mp-7443	Cr	$NbSe_2$	-2.28	1.96	2.26	3D : 0.996
mp-985289	Cr	$NbSe_2$	-2.08	2.6	2.04	3D : 1.0
mp-1193575	Mn	$NbSe_2$	-2.09	2.63	2.07	3D : 0.992
mp-1190037	Fe	$NbSe_2$	-1.49	2.76	2.04	3D : 0.986
mp-1193638	Fe	$NbSe_2$	-1.2	2.73	2.07	3D : 0.987
mp-1186227	Co	$NbSe_2$	-1.03	2.83	2.11	3D : 0.963
mp-1193368	Co	$NbSe_2$	-0.751	2.68	2.07	3D : 0.962
mp-20279	In	$NbSe_2$	-0.917	2.8	2.07	3D : 0.534
mp-1018116	In	$NbSe_2$	-1.38	2.92	2.05	02D : 0.712
mp-755664	Li	TaS_2	-1.48	3.19	2.21	3D : 0.754
mp-1206881	Li	TaS_2	-1.85	2.78	2.26	3D : 0.773
mp-1222716	Li	TaS_2	-1.63	2.98	2.26	2D : 0.683
mp-1221434	Na	TaS_2	-1.48	3.34	2.26	3D : 0.917
mp-1223710	K	TaS_2	-1.79	3.3	2.26	3D : 0.991
mp-1190092	V	TaS_2	-1.28	3.24	2.26	3D : 1.0
mp-1189011	Cr	TaS_2	-1.44	3.11	2.37	3D : 1.0
mp-3581	Mn	TaS_2	-1.63	2.8	2.22	3D : 0.989
mp-1208432	Mn	TaS_2	-1.79	2.96	2.22	3D : 1.0
mp-1218077	Ni	TaS_2	-1.48	2.15	2.22	2D : 0.968
mp-1218136	Ni	TaS_2	-1.46	2.4	2.26	2D : 0.919
mp-1219081	Ni, Cr	TaS_2	-1.51	2.08	2.22	2D : 0.966
mp-554416	Fe	TaS_2	-0.752	2.88	2.21	3D : 0.987
mp-1218020	Fe	TaS_2	-2.24	2.59	2.22	2D : 0.959
mp-1218051	Mo	TaS_2	-1.7	2.21	2.26	2D : 0.965
mp-22332	In	TaS_2	-1.05	3.12	2.28	02D : 0.528
mp-1101055	In	TaS_2	-1.47	2.92	2.24	02D : 0.551
mp-1218110	In	TaS_2	-1.15	2.86	2.24	02D : 0.591
mp-1218117	In	TaS_2	-1.27	3.03	2.22	02D : 0.591
mp-1221424	Na	$TaSe_2$	-1.8	2.35	2.44	3D : 0.946
mp-1223811	K	$TaSe_2$	-2.93	1.72	2.49	2D : 0.981
mp-1188354	V	$TaSe_2$	-2.65	1.86	2.44	3D : 1.0
mp-1187280	Cr	$TaSe_2$	-2.73	1.77	2.44	3D : 1.0
mp-1208377	Cr	$TaSe_2$	-2.35	2.19	2.47	3D : 1.0
mp-999136	In	$TaSe_2$	-1.09	2.72	2.45	02D : 0.507
mp-1208590	In	$TaSe_2$	-1.43	2.56	2.48	02D : 0.681
mp-1218192	In	$TaSe_2$	-1.42	2.51	2.48	02D : 0.702
mp-30533	K	Pt_2S_3	-0.836	1.71	1.62	3D : 0.933
mp-28987	Na	Pt_2Se_3	-1.25	1.67	2.13	3D : 0.731
mp-14796	K	Pt_2Se_3	-1.02	1.68	2.14	3D : 0.83
mp-14797	Rb	Pt_2Se_3	-0.939	1.68	2.13	3D : 0.977
mp-573316	Cs	Pt_2Se_3	-0.96	1.68	2.14	3D : 1.0
mp-1247173	Mg, Al	Mn_3S_8	-1.31	1.15	0.854	3D : 0.792
mp-1384478	Mn, Zn	Mn_3S_8	-1.08	0.902	0.854	3D : 0.714
mp-1410942	Mn, Zn	Mn_3S_8	-0.976	0.789	0.852	3D : 0.6
mp-1443978	Mn, Mg	Mn_3S_8	-0.834	0.965	0.855	3D : 0.94
mp-17228	K	Ni_3S_4	-1.28	0.934	1.23	3D : 0.799
mp-672177	K	Ni_3S_4	-1.17	0.885	1.23	3D : 0.86

mp-1079718	Rb	Ni_3S_4	-1.2	1.05	1.23	3D : 0.915
mp-28486	Cs	Ni_3S_4	-1.12	1.14	1.23	3D : 0.994
mp-1080141	Cs	Ni_3S_4	-1.2	1.1	1.23	3D : 1.0
mp-9910	K	Pd_3S_4	-1.11	1.51	1.11	3D : 0.823
mp-11695	Rb	Pd_3S_4	-1.11	1.48	1.08	3D : 0.918
mp-663190	Rb	Pd_3S_4	-1.06	1.51	1.2	3D : 0.946
mp-1205388	Rb	Pd_3S_4	-1.11	1.49	1.09	3D : 0.924
mp-510268	Cs	Pd_3S_4	-1.12	1.48	1.09	3D : 0.995
mp-14339	K	Pd_3Se_4	-1.01	1.26	1.3	3D : 0.739
mp-683041	K	Pd_3Se_4	-0.917	1.34	1.53	3D : 0.717
mp-14340	Rb	Pd_3Se_4	-0.929	1.27	1.32	3D : 0.852
mp-683059	Rb	Pd_3Se_4	-0.817	1.31	1.52	3D : 0.858
mp-11694	Cs	Pd_3Se_4	-0.902	1.3	1.3	3D : 0.967
mp-4030	Rb	Pt_3S_4	-1.09	1.41	1.47	3D : 0.907
mp-663224	Rb	Pt_3S_4	-1.03	1.4	1.59	3D : 0.918
mp-13992	Cs	Pt_3S_4	-1.08	1.43	1.46	3D : 0.995
mp-14338	Cs	Pt_3Se_4	-0.937	1.27	1.88	3D : 0.968
mp-1079772	Ca	InP	-3.2	0.816	0.779	3D : 0.894
mp-1078973	Sr	InP	-3.41	0.843	0.793	3D : 0.866
mp-1078002	Mn	$GaSe_2$	-0.822	1.29	1.39	3D : 0.999
mp-1199743	Cs	$InTe_2$	-0.939	0.919	1.22	3D : 0.939
mp-505005	Ce	OsSi	-4.9	1.56	1.81	3D : 1.0
mp-754516	Li	NiP	-1.41	1.16	1.04	3D : 0.716
mp-4767	Ce	OsSi	-4.26	1.45	1.81	3D : 1.0
mp-1206349	K	CoP	-1.97	1.19	1.93	3D : 0.808
mp-9473	Ba	NiP	-3.02	1.01	1.05	3D : 0.903
mp-1206920	K	IrP	-1.8	1.22	1.56	3D : 0.667
mp-1206529	Rb	IrP	-1.88	1.31	1.55	3D : 0.786
mp-4815	Pr	RhSi	-4.42	2.38	2.56	3D : 1.0
mp-10698	Ba	RhGe	-3.38	1.78	2.02	3D : 0.932
mp-1207113	Rb	RhP	-2.06	1.5	1.59	3D : 0.785
mp-567408	Sm	OsSi	-4.19	1.29	1.81	3D : 1.0
mp-1206405	Cs	RhP	-2.18	1.51	1.59	3D : 0.928
mp-11169	Ba	IrP	-3.93	1.09	1.56	3D : 0.878
mp-571586	Nd	OsSi	-4.25	1.27	1.81	3D : 1.0
mp-567203	La	OsSi	-4.31	1.32	1.81	3D : 1.0
mp-1192652	La, Al	OsB	-3.41	1.52	5.05	3D : 1.0
mp-978853	Sr	IrGe	-3.21	1.23	1.77	3D : 0.985
mp-5936	La	RhSi	-4.4	2.37	2.56	3D : 1.0
mp-12098	K	RhP	-1.99	1.44	1.59	3D : 0.68
mp-3585	La	IrSi	-3.94	2.25	1.84	3D : 1.0
mp-5852	Pr	OsSi	-4.4	1.29	1.81	3D : 1.0
mp-12073	Ba	IrB	-3.74	1.01	0.866	3D : 1.0
mp-1206424	Sr	PtSi	-3.69	1.86	1.14	3D : 0.997
mp-21849	Eu	IrSi	-3.43	2.15	1.84	3D : 1.0
mp-1207365	Cs	IrP	-2.02	1.26	1.56	3D : 0.932
mp-21383	Eu	RhSi	-3.77	2.29	2.56	3D : 1.0
mp-10697	Sr	RhGe	-3.47	1.69	2.02	3D : 0.987
mp-8581	Sr	RhP	-3.9	1.29	1.61	3D : 0.849
mp-1206941	Rb	CoP	-2.24	1.23	1.93	3D : 0.889
mp-8583	Ba	RhP	-4.12	1.37	1.62	3D : 0.89
mp-8982	Ca	PtSi	-3.49	1.88	2.07	3D : 1.0
mp-20615	Eu	PdGe	-3.55	1.46	1.23	3D : 1.0
mp-978253	Ce	RhSi	-4.79	2.34	2.53	3D : 0.988
mp-1193516	Eu	PdSn	-3.89	1.41	1.05	3D : 1.0
mp-504772	Y	RhSi	-4.83	2.32	2.53	3D : 1.0
mp-1211651	La	RhGe	-4.9	1.55	1.43	3D : 1.0
mp-1208977	Sm	RhSi	-4.55	2.21	2.53	3D : 1.0
mp-1208966	Sm	PdSi	-4.45	2.02	1.41	3D : 0.965
mp-627355	Ce	PtGe	-3.9	1.98	1.1	3D : 1.0
mp-1215178	Zr	TiB_4	-2.06	0.867	1.26	3D : 1.0
mp-1224263	Hf	TiB_4	-2.04	0.787	1.25	3D : 1.0
mp-1215211	Zr	NbB_4	-2.44	1.56	2.54	3D : 1.0

mp-1224328	Hf	NbB_4	-2.59	1.48	2.55	3D : 1.0
mp-1215209	Zr	TaB_4	-2.72	1.5	2.5	3D : 1.0
mp-1224283	Hf	TaB_4	-2.65	1.46	2.52	3D : 1.0
mp-1220697	Al	Nb_2B_6	-4.9	0.772	1.45	02D : 0.532
mp-996161	Al	Nb_3C_2	-3.2	1.13	1.36	3D : 0.997
mp-569568	Al	Ta_3C_2	-2.65	1.11	1.06	3D : 1.0
mp-1216678	Ti	MoP_2	-1.07	1.65	1.15	3D : 1.0
mp-1216676	Ti	WP_2	-0.924	0.951	0.78	3D : 1.0
mp-1104130	Ba	AuP_2	-2.55	0.824	0.969	3D : 0.948
mp-1246556	Sr	VN_2	-3.57	1.66	0.814	3D : 1.0
mp-1029711	Na	VN_2	-1.22	1.74	0.815	3D : 1.0
mp-1246136	Ba	VN_2	-4.6	1.28	0.816	3D : 1.0
mp-1245629	Ba	VN_2	-3.85	1.85	0.816	3D : 1.0
mp-752942	Li	TiO_2	-1.1	1.83	2.62	3D : 0.993
mp-780233	Li	TiO_2	-1.12	1.01	2.63	3D : 1.0
mp-1387047	Ca	TiO_2	-1.64	1.52	2.64	3D : 1.0
mp-760000	Li, V	TiO_2	-1.2	0.774	2.68	3D : 0.992
mp-757308	Na	TiO_2	-1.46	1.27	2.64	3D : 1.0
mp-1101470	Na	TiO_2	-1.72	1.58	2.79	3D : 1.0
mp-768342	Sr	NbO_2	-2.86	2.64	0.876	3D : 1.0
mp-867955	Na	NbO_2	-0.854	2.31	0.752	3D : 0.989
mp-29792	Ca	NbO_2	-1.99	2.78	0.93	3D : 1.0
mp-31908	Mn	Nb_6O_{11}	-2.12	2.52	1.76	3D : 0.943
mp-760837	Rb	$Nb_{10}O_{17}$	-1.02	2.51	2.46	3D : 1.0
mp-8236	Ba	PdP	-3.12	0.931	1.38	3D : 0.993
mp-1227860	Ba	PdP	-3.39	1.39	1.24	3D : 1.0
mp-28339	Ca	PtP	-2.33	1.85	3.1	3D : 0.919
mp-1218299	Eu, Sr	PtP	-2.32	1.62	0.817	3D : 1.0
mp-685613	Ba	Pd_2P	-4.3	1.09	1.31	3D : 1.0
mp-504701	Cs	TiF_4	-1.45	2.5	1.04	3D : 1.0
mp-21639	K	TiF_4	-0.949	2.52	1.13	3D : 0.965
mp-1120745	Na	TiF_4	-0.947	2.52	1.07	3D : 1.0
mp-27264	Na	TiF_4	-0.996	2.48	1.16	3D : 1.0
mp-754779	Li	NbS_3	-0.959	2.86	1.46	3D : 0.638
mp-769050	Li	NbS_3	-0.9	2.62	1.47	3D : 0.636
mp-2492941	Li	NbS_3	-0.907	2.62	1.48	3D : 0.616
mp-570823	Ba	BSe_3	-1.85	1.0	0.893	2D : 0.509
mp-30105	Ba	B_4Se_{13}	-1.72	0.929	1.3	3D : 0.986
mp-28463	Li	Nb_3Cl_8	-0.815	2.35	1.65	3D : 0.935

TABLE S5: Best candidates binary etched layers. The values of the highest etched energy (HEE), lowest non-etched energy (LNE) and lowest defect formation energy (LDFE) are given in eV.

MP ID	Etched	Layer	HEE (eV)	LNE (eV)	LDFE (eV)	Initial Dim.
mp-1226508	Ce	$OsRuSi_2$	-4.03	0.534	0.692	3D : 1.0
mp-1220305	Nd	$RuRhSi_2$	-3.79	0.502	0.597	3D : 1.0
mp-1225172	Eu	Co_2GeSi	-2.98	0.594	0.731	3D : 1.0
mp-7129	Rb	$TiCu_2S_4$	-1.2	0.795	1.28	3D : 0.968
mp-10489	Cs	$TiCu_2Se_4$	-1.2	0.585	1.5	3D : 0.978
mp-10488	Cs	$TiAg_2S_4$	-0.992	0.541	1.18	3D : 0.948
mp-1220884	Na	$TiVS_4$	-0.984	1.59	1.44	3D : 0.899
mp-1222735	Li	$CrVS_4$	-1.22	0.951	1.22	3D : 0.644
mp-1223464	K	$CrSnS_4$	-0.841	1.54	1.13	3D : 0.923
mp-2222797	Mg	Nb_2PdS_6	-3.05	1.65	1.16	3D : 0.923
mp-1228980	Al	$CuGeSe_4$	-2.06	0.889	0.674	3D : 0.992
mp-1228963	Al	$AgSnSe_4$	-1.84	0.741	0.609	3D : 0.992
mp-1220518	Ni	Nb_2CS_2	-0.624	1.37	1.4	3D : 0.966
mp-1218138	Ni	Ta_2CS_2	-0.926	1.62	1.71	3D : 0.901
mp-1195350	Cs	$CdInTe_3$	-0.872	0.723	1.38	3D : 0.892
mp-541407	Rb	$CuSnS_3$	-0.655	1.18	1.11	3D : 0.809
mp-21713	K	$CuIn_3Se_6$	-0.738	0.697	1.14	3D : 0.791
mp-680403	K	$AgIn_3Se_6$	-0.649	0.662	1.13	3D : 0.956
mp-6376	K	VCu_2S_4	-0.533	0.967	1.47	3D : 0.646
mp-10091	K	VCu_2Se_4	-0.643	0.792	1.07	3D : 0.679
mp-1106233	K	$NbCu_2S_4$	-0.629	0.691	1.17	3D : 0.56
mp-6599	K	$NbCu_2Se_4$	-0.7	0.656	0.915	3D : 0.642
mp-505321	Cs	$NbCu_2Te_4$	-0.613	0.685	0.813	3D : 0.948
mp-6013	K	$TaCu_2Se_4$	-0.687	0.668	1.04	3D : 0.623
mp-505322	Cs	$TaCu_2Te_4$	-0.723	0.599	0.78	3D : 0.939
mp-571288	K	$TaAg_2Se_4$	-0.716	0.53	0.855	3D : 0.474
mp-1192531	K	Cu_2SbS_3	-0.739	0.575	1.05	3D : 0.94
mp-1194436	K	Cu_2BiS_3	-0.735	0.564	1.15	3D : 0.95
mp-1176770	Li	CrP_2S_7	-1.34	1.02	0.836	02D : 0.787
mp-510569	Cs	$CeCuS_3$	-0.712	0.879	1.3	3D : 0.946
mp-505171	Na	$TiCuS_3$	-0.881	0.703	1.18	3D : 0.73
mp-1220939	Na	TiNCl	-0.798	2.7	2.45	3D : 0.594
mp-679669	Na	Zr_2N_2ClS	-1.32	3.11	1.09	3D : 0.947
mp-1220698	Na	Zr_2N_2ClS	-1.12	2.73	0.774	3D : 0.749

TABLE S6. Best candidates ternary layers. The values of the highest etched energy (HEE), lowest non-etched energy (LNE) and lowest defect formation energy (LDFE) are given in eV.

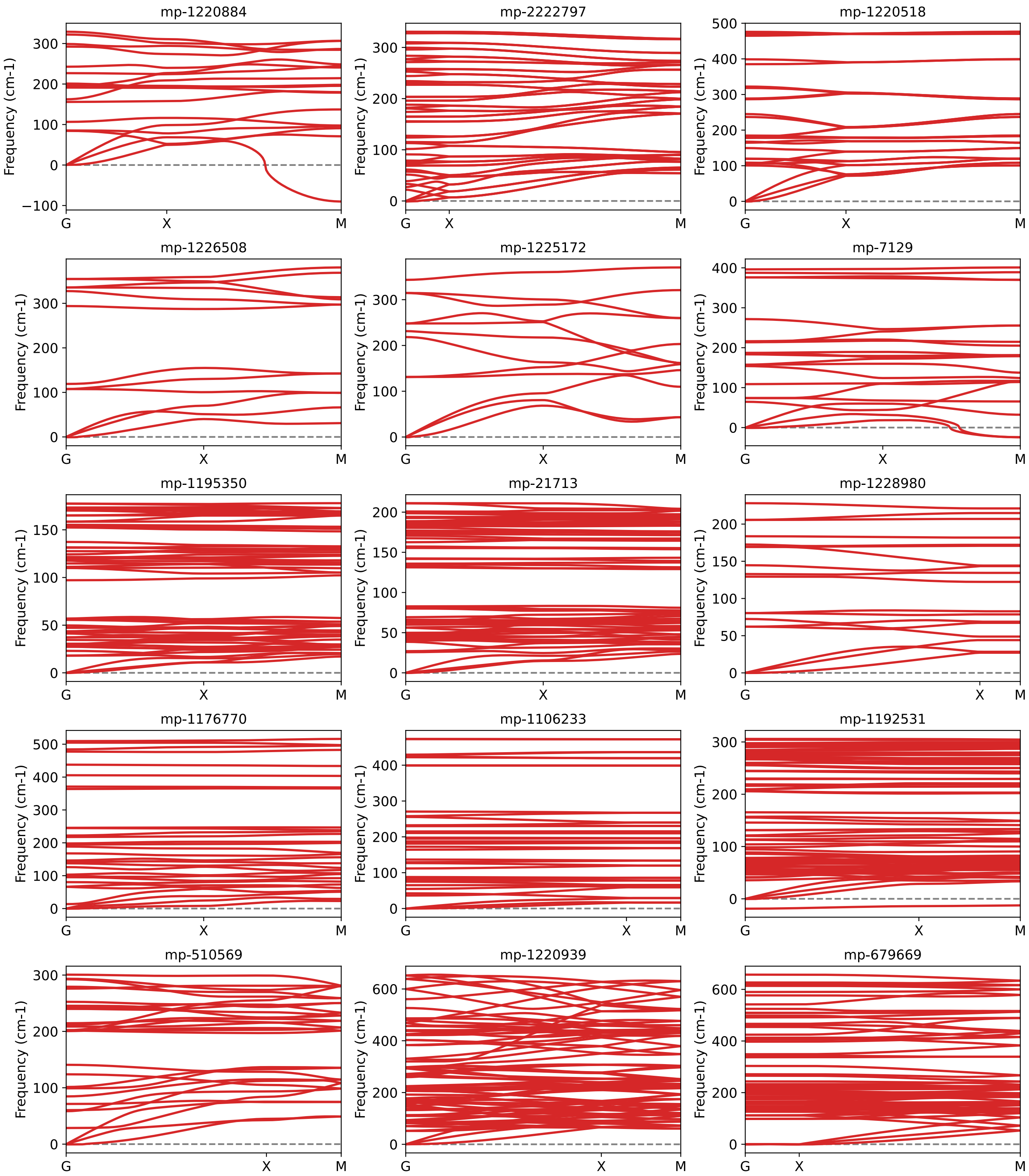

FIG. S11. Phonon dispersion curves for the ternary candidates presented in Fig. 6 of the main text.

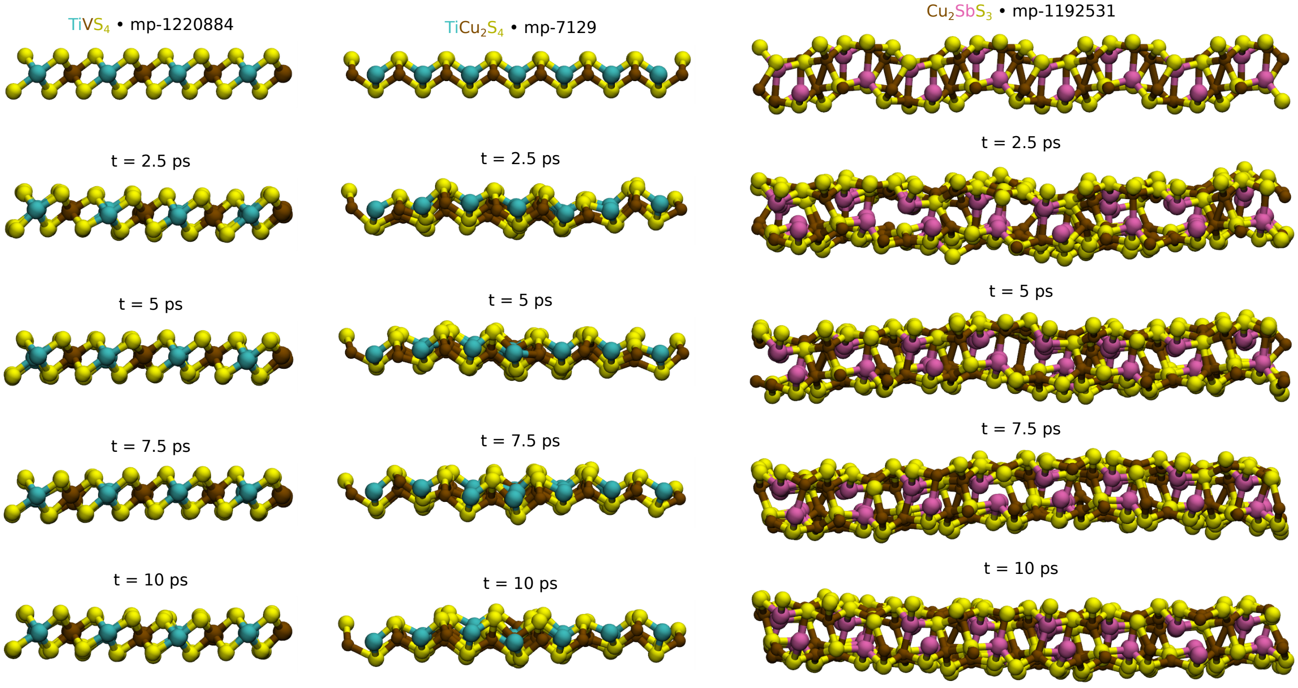

FIG. S12. MD snapshots of the 3 layers showing negative phonon frequencies in Fig. S11.

V. CHEMICAL POTENTIALS

Two sets of chemical potentials are used in the main text: the elemental ones μ^E and the chemical potential in HF μ^{HF}. The former is obtained from the structures with lowest formation energies available in the MP database. Note that the potentials μ^{E} are then computed using all four UMLIPs. A comparison between UMLIPs and DFT is presented in Fig. S13. While MACE, CHGNet and M3GNet lead to extremely accurate atomic energies, ALIGNN fails for some elements, resulting in a much higher RMSE. This could in part explain the less accurate predictions of the vacancy formation energies presented in Fig. 1 of the main text.

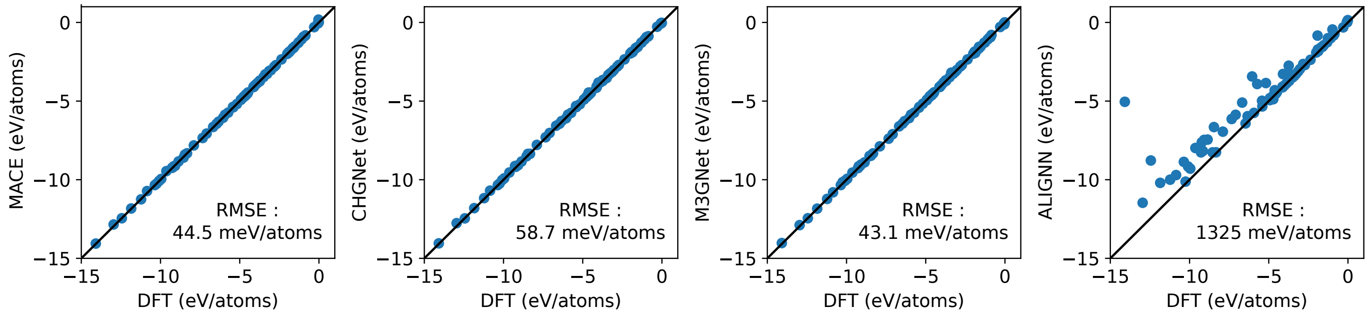

FIG. S13. Comparison of the atomic formation energy from DFT calculations with different UMLIPs. Reference DFT values are taken from the Materials Project database.

For chemical potentials in HF, we used the ones previously used in Ref. 4. Additionally, we also included potentials of lanthanides using the same method. All chemical potentials are presented in Tables S7 and S8 below. Note that for simplicity, the table contains the value $\mu^{\mathrm{HF}} - \mu^{\mathrm{E}}$ rather than μ^{HF}.

Element	μ^{E} from MP	μ^{E} from MACE	$\mu^{\mathrm{HF}} - \mu^{\mathrm{E}}$
H	-3.39	-3.30	0.00
Li	-1.90	-1.91	-3.22
Be	-3.74	-3.75	-4.24
B	-6.68	-6.65	0.00
C	-9.23	-9.21	0.00
N	-8.33	-8.32	0.00
O	-4.92	-4.90	-2.46
F	-1.87	-1.87	-3.00
Na	-1.31	-1.31	-2.89
Mg	-1.59	-1.61	-4.89
Al	-3.74	-3.71	-5.84
Si	-5.42	-5.37	0.00
P	-5.41	-5.40	0.00
S	-4.14	-4.11	0.00
Cl	-1.84	-1.84	0.00
K	-1.09	-1.07	-3.12
Ca	-2.00	-2.00	-5.92
Sc	-6.33	-6.31	-6.26
Ti	-7.90	-7.82	-3.80
V	-9.08	-9.12	-2.69
Cr	-9.63	-9.45	-2.32
Mn	-9.16	-9.14	-2.54
Fe	-8.46	-8.40	-1.13
Co	-7.09	-7.07	-0.74
Ni	-5.73	-5.73	-0.65
Cu	-4.10	-4.09	0.00
Zn	-1.26	-1.25	-1.70
Ga	-3.03	-3.04	-2.11
Ge	-4.62	-4.60	0.00
Se	-3.49	-3.47	0.00
Br	-1.64	-1.62	-0.07

TABLE S7. Chemical potentials for elements from H to Cl. Both the elemental potentials and the potentials in HF are presented.

Element	μ^{E} from MP	μ^{E} from MACE	$\mu^{HF} - \mu^{E}$
Rb	-0.98	-0.91	-3.12
Sr	-1.68	-1.68	-5.98
Y	-6.46	-6.45	-7.37
Zr	-8.54	-8.59	-5.96
Nb	-10.09	-10.08	0.00
Mo	-10.85	-10.72	0.00
Ru	-9.28	-9.25	-1.38
Rh	-7.34	-7.35	0.00
Pd	-5.18	-5.19	0.00
Ag	-2.83	-2.82	0.00
Cd	-0.91	-0.88	-0.98
In	-2.72	-2.71	-1.39
Sn	-4.01	-3.97	-0.65
Sb	-4.12	-4.07	0.00
Te	-3.14	-3.09	0.00
I	-1.52	-1.52	-0.00
Cs	-0.85	-0.82	-3.20
Ba	-1.92	-1.91	-5.99
La	-4.93	-4.92	-7.26
Ce	-5.93	-5.87	-7.14
Pr	-4.77	-4.76	-7.22
Nd	-4.76	-4.74	-7.14
Sm	-4.71	-4.70	-7.09
Eu	-10.25	-10.25	-6.13
Gd	-14.09	-14.06	-7.03
Tb	-4.62	-4.61	-6.93
Dy	-4.59	-4.59	-7.07
Ho	-4.58	-4.56	-7.16
Er	-4.57	-4.56	-7.11
Tm	-4.47	-4.45	-7.04
Lu	-4.51	-4.52	-6.83
Hf	-9.96	-9.95	-5.93
Ta	-11.85	-11.83	0.00
W	-12.96	-12.85	0.00
Re	-12.44	-12.45	-0.52
Os	-11.22	-11.25	0.00
Ir	-8.86	-8.84	0.00
Pt	-6.05	-6.06	0.00
Au	-3.27	-3.27	0.00
Hg	-0.30	-0.30	0.00
Tl	-2.37	-2.35	-0.51
Pb	-3.71	-3.69	-0.43
Bi	-3.88	-3.84	0.00

TABLE S8. Chemical potentials for elements from K to Bi. Both the elemental potentials and the potentials in HF are presented.

VI. UMLIP MODELS IN ASE

For MACE, we use the pre-trained MACE-MP-0 model, with small model size and withouth dispersion. It is directly loaded from the mace python package using the following code:

```
from mace.calculators import mace_mp
MACE = mace_mp(model="small", dispersion=False, default_dtype="float32", device='cuda')
```

For CHGNet, we use the default model from the chgnet python package, which can be loaded as:

```
from chgnet.model.dynamics import CHGNetCalculator
CHGNet = CHGNetCalculator(use_device='cuda', on_isolated_atoms='ignore')
```

For M3GNet, we use the model trained on MP and it is loaded using the matgl package.

```
import matgl
from matgl.ext.ase import PESCalculator
M3GNet = PESCalculator(matgl.load_model("M3GNet-MP-2021.2.8-PES"))
```

Similarly, the ALIGNN model trained on MP can be loaded using the alignn python package.

```
from alignn.ff.ff import AlignnAtomwiseCalculator, mptraj_path
ALIGNN = AlignnAtomwiseCalculator(path=mptraj_path())
```


[1] T. Angsten, T. Mayeshiba, H. Wu, and D. Morgan, "Elemental vacancy diffusion database from high- throughput first-principles calculations for fcc and hcp structures," (2014).
[2] T. Angsten, T. Mayeshiba, H. Wu, and D. Morgan, New Journal of Physics **16**, 015018 (2014).
[3] P. Huang, R. Lukin, M. Faleev, N. Kazeev, A. R. Al-Maeeni, D. V. Andreeva, A. Ustyuzhanin, A. Tormasov, A. H. Castro Neto, and K. S. Novoselov, npj 2D Materials and Applications **7**, 6 (2023).
[4] J. Björk, J. Zhou, P. O. A. Persson, and J. Rosen, Science **383**, 1210 (2024).
[5] J. Davidsson, F. F. Bertoldo, K. S. Thygesen, and R. Armiento, "Impurities in 2D Materials Database," (2022).
[6] M. H. Rahman, P. Gollapalli, P. Manganaris, S. K. Yadav, G. Pilania, B. DeCost, K. Choudhary, and A. Mannodi-Kanakkithodi, APL Machine Learning **2**, 016122 (2024).
